# Higher blood pressure versus normotension targets to prevent acute kidney injury: a systematic review and meta-regression of randomized controlled trials

**DOI:** 10.1186/s13054-022-04236-1

**Published:** 2022-11-25

**Authors:** Phu Nguyen Trong Tran, Prit Kusirisin, Piyanut Kaewdoungtien, Jeerath Phannajit, Nattachai Srisawat

**Affiliations:** 1 Division of Nephrology, Department of Medicine, Faculty of Medicine, Chulalongkorn University, Bangkok, Thailand; 2 Excellence Center for Critical Care Nephrology, King Chulalongkorn Memorial Hospital, Bangkok, Thailand; 3 Center of Excellence in Critical Care Nephrology, Faculty of Medicine, Chulalongkorn University, Bangkok, Thailand; 4 Division of Nephrology, Department of Internal Medicine, Faculty of Medicine, Chiang Mai University, Chiang Mai, Thailand; 5 Division of Clinical Epidemiology, Department of Medicine, Faculty of Medicine, Chulalongkorn University, Bangkok, Thailand; 6 Division of Nephrology, Police General Hospital, Royal Thai Police Headquarters, Bangkok, Thailand; 7 Academy of Science, Royal Society of Thailand, Bangkok, Thailand; 8 Department of Internal Medicine, Faculty of Medicine, Can Tho University of Medicine and Pharmacy, Cantho, Vietnam

**Keywords:** Shock, Perioperative, Mean arterial blood pressure, Acute kidney injury, Renal replacement therapy, Hypertension

## Abstract

**Background:**

Renal hypoperfusion is one of the most common causes of acute kidney injury (AKI), especially in shock and perioperative patients. An optimal blood pressure (BP) target to prevent AKI remains undetermined. We conducted a systematic review and meta-analysis of available randomized clinical trial (RCT) results to address this knowledge gap.

**Methods:**

From inception to May 13, 2022, we searched Ovid Medline, EMBASE, Cochrane Library, SCOPUS, clinicaltrials.gov, and WHO ICTRP for RCTs comparing higher BP target versus normotension in hemodynamically unstable patients (shock, post-cardiac arrest, or surgery patients). The outcomes of interest were post-intervention AKI rate and renal replacement therapy (RRT) rate. Two investigators independently screened the citations and reviewed the full texts for eligible studies according to a predefined form.

**Results:**

Twelve trials were included, enrolling a total of 5759 participants, with shock, non-cardiac, and cardiac surgery patients accounting for 3282 (57.0%), 1687 (29.3%) and 790 (13.7%) patients, respectively. Compared to lower mean arterial blood pressure (MAP) targets that served as normotension, targeting higher MAP had no significant effect on AKI rates in shock (RR [95% CI] = 1.10 [0.93, 1.29]), in cardiac-surgery (RR [95% CI] = 0.87 [0.73, 1.03]) and non-cardiac surgery patients (RR [95% CI] = 1.25 [0.98, 1.60]) using random-effects meta-analyses. In shock patients with premorbid hypertension, however, targeting MAP above 70 mmHg resulted in significantly lower RRT risks, RR [95%CI] = 1.20 [1.03, 1.41], p < 0.05.

**Conclusions:**

Targeting a higher MAP in shock or perioperative patients may not be superior to normotension, except in shock patients with premorbid hypertension. Further studies are needed to assess the effects of a high MAP target to preventing AKI in hypertensive patients across common settings of hemodynamic instability.

*Trial registration* This systematic review has been registered on PROSPERO (CRD42021286203) on November 19, 2021, prior to data extraction and analysis.

**Supplementary Information:**

The online version contains supplementary material available at 10.1186/s13054-022-04236-1.

## Introduction

Acute kidney injury (AKI) is a global health burden, with an incidence of roughly 13.3 million cases per year, contributing to approximately 1.7 million deaths [1, 2]. AKI is linked to higher morbidity, death, and expenses.

AKI is a multicausal syndrome with complex pathophysiology, making the development of an effective treatment a challenging research area. To date, no effective pharmacologic therapy has been established to counteract the disorder. Consequently, according to the Kidney Disease Improving Global Outcome (KDIGO) AKI guidelines [3], management of AKI should focus on implementing interventions to prevent its development, providing supportive care to prevent further injury, facilitate renal recovery and treat complications.

Renal hypoperfusion is one of the most common causes of AKI, especially in shock- and surgery-related AKI patients. Optimizing hemodynamics therefore is essential for the prevention and treatment of AKI in these settings. Among the strategies, maintaining a physiological MAP is necessary to ensure sufficient kidney perfusion pressure and microcirculatory blood flow [26]. However, there were still conflicting results regarding the benefit of high MAP to renal outcomes [4, 5].

Given the higher exposure to fluid and vasopressor use of higher MAP and the heterogeneous settings of AKI patients, it is essential to investigate: (1) whether higher MAP levels, compared to normotension, should be considered to prevent AKI, and (2) in which AKI settings higher MAP are more likely to be beneficial. We thus conducted a comprehensive review with meta-analysis based on randomized controlled trials (RCT) that examined the effect of high versus normotensive MAP on AKI incidence or progression across common settings (shock, cardiac or non-cardiac surgery), and used meta-regression to examine several characteristics that may serve as effect-modifiers.

## Materials and methods

We conducted a systematic review with meta-analysis and meta-regression. The protocol for this systematic review was registered on PROSPERO (CRD42021286203) on November 19, 2021, prior to data extraction and analysis. We reported our study results in accordance with PRISMA guideline.

### Systematic search

From inception until May 13, 2022, we searched MEDLINE, EMBASE, the Cochrane Library, and SCOPUS for RCTs comparing higher and lower blood pressure (BP) target in shock or perioperative patients. We also searched Clinicaltrials.gov and WHO ICTRP for relevant trial registries. We built the search queries using three concepts: (1) shock, post-cardiac arrest, perioperative patients (2) blood pressure target, and (3) randomized controlled trial. We used both the Medical Subject Headings database and free-text syntax without any language restrictions (Additional file 1: Appendix 1–6). We further performed reference screening on the included studies for other eligible trials.

### Study selection

We included studies that met all of the following criteria: RCT with two or more arms targeting higher BP as compared to lower BP targets, which serve as normotension level; targeting BP levels were the sole hemodynamic strategy that were intended using vasopressors; studies which reported renal outcomes such as incidence or rates of AKI any stage (according to KDIGO [3], RIFLE [6], AKIN [7] criteria or other equivalent definitions) or rates of renal replacement therapy (RRT) receipt. For outcomes reported at multiple timepoints, we used the furthest reported follow-up timepoint but capped at 30-day timepoints. We excluded studies that met any of the following criteria: (1) animal research and (2) pediatrics (less than 15 years of age) or obstetrics research.

### Study screening

We used COVIDENCE to remove duplicates and screen citations following three steps prior to data extraction: (1) screening of titles and abstracts, (2) searching for full texts and results, and (3) reviewing the full texts (Additional file 1: Appendix 7). After duplicate removal, two investigators (Tran NTP and Kusirisin P) independently screened the citations. Conflicts were resolved by consensus with the third investigator (Kaewdoungtien P). For citations with no available full texts, we contacted the corresponding authors by email and ResearchGate direct messages. After all attempts, those whose full texts and results were not available were classified as “Studies awaiting classification”, or “Studies ongoing” if the trials were not completed (Additional file 1: Appendix 8–10). Finally, two investigators (Tran NTP and Kusirisin P) independently reviewed the studies for eligibility and captured reasons for exclusion at this step.

### Data extraction and risk of bias assessment

Reviewers independently extracted data using a predefined data abstraction excel form. We extracted the following information: study title, first author, year of publication, funding, setting of recruitment sites, characteristics of the population (studied conditions, age, sex, premorbid hypertension), intervention (BP targets, time of intervention, protocol used to reach the targets), AKI and RRT receipt rate. We contacted the authors via email for further information or unreported results. Some unavailable original data (hypertension percentages) were imputed using the percentage of anti-hypertension medication.

We assessed risk of bias (RoB) independently using Cochrane RoB updated version (RoB-2) for which each domain is rated as “low risk”, “high risk”, or “some concerns”. We assessed RoB-2 according to intention-to-treat basis, based on one main outcome of interest reported in the studies: AKI rates and RRT receipt rates, respectively.

We assessed the certainty of the body of evidence for each outcome by the Grading Recommendations Assessment, Development and Evaluation (GRADE) approach. We used the Guideline Development Tool (https://www.gradepro.org) to formulate the Summary of Findings table. Any disagreements regarding RoB or GRADE assessment were resolved by consensus.

### Data conversion and preparation for synthesis

Normotensive levels were determined according to the common “lower” MAP intervals across the patient populations. Where the BP targets were not available numerically (for instance, “usual care” or “standard care” arm), we used the average BP within the intervention period. Since some studies reported AKI using indirect definitions, we did some conversions (Additional file 1: Appendix 11).

### Statistical analysis

We conducted meta-analysis on outcomes that were reported in at least two studies. We employed the risk ratio (RR) as the main effect size estimate for dichotomous variables (rates of AKI and RRT receipt) all with 95% confidence interval and *p* value, using the Mantel–Haenszel statistical method. A significant difference was defined as *p* < 0.05. We performed all meta-analyses with random-effects models and by Revman version 5.4 (Cochrane Collaboration, Oxford) software.

We assessed heterogeneity between trials by visual inspection of the forest plots, the chi-squared test for homogeneity (where *p* < 0.1 indicates important heterogeneity), and the *I*^2^ statistic. We did not conduct meta-analyses where I^2^ indicated considerable heterogeneity (*I*^2^ ≥ 75%).

We conducted meta-analysis on the predefined groups of different populations: (1) shock, (2) cardiac surgery and (3) non-cardiac surgery patients. We also conducted subgroup analysis based on the discrepancy level between normotensive and high MAP, and on premorbid hypertension condition where reported results were available. To detect publication bias, we performed funnel plots for primary outcome synthesis and inspected for any asymmetry.

We performed meta-regression using comprehensive meta-analysis version 3 to examine the relationship between predefined potential moderators: (1) percentage of hypertension patients in the cohort, (2) mean age and (3) risk of bias level and the treatment effect size (Mantel–Haenszel log risk ratio). All meta-regression models were adjusted for study group (i.e. group of shock, cardiac surgery or non-cardiac surgery). Q model statistics with *p* value < 0.05 indicate that the relationship between moderator variables and effect size is stronger than we would expect by chance. Moderators with Z statistics with *p* < 0.05 were interpreted as their slope is probably not zero, and the treatment effect size would vary according to changes in moderator variables. Positive value of MH log risk ratio is in favour of the renoprotective effect of high MAP.

We did not conduct trial sequential analysis due to the anticipated heterogeneity of the included populations.

## Results

**Fig. 1:**
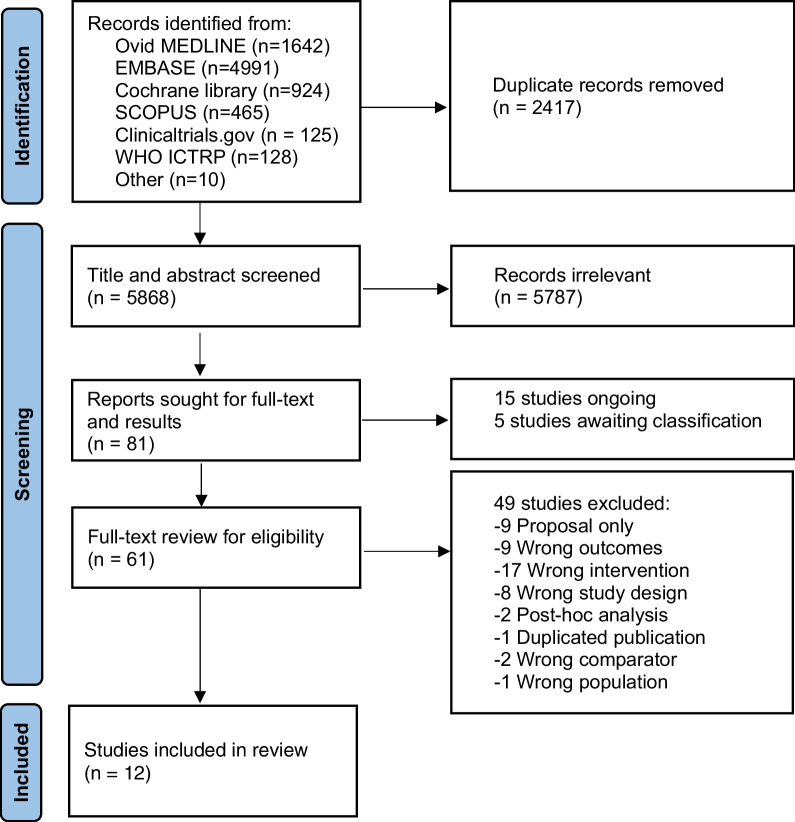
Flow diagram of citation selection

We retrieved a total of 8285 citations and removed 2417 duplicates (Fig. 1). We then excluded 5787 irrelevant studies in the title and abstract screening step. After full-text and result seeking, we classified 15 as “ongoing” and 5 as “awaiting classification”. In the full-text assessment, we further excluded 49 studies, leaving 12 studies for our review (Fig. 1).

### Description of the included studies

Among the included studies, 6 were multi-center RCTs and 6 were single center (Additional file 1: Appendix 11a). Only one RCT was able to conduct on a double-blinded basis [8]. Ten studies were conducted in Europe, two in China.

We grouped the included studies based on their recruited populations: shock (3 studies) [8–10], non-cardiac surgery (4 studies) [11–14] and cardiac surgery (5 studies) [15–19] (Table 1). Among the 5759 total patients, shock, non-cardiac and cardiac surgery accounted for 3282 (57.0%), 1687 (29.3%) and 790 (13.7%) patients, respectively (Additional file 1: Appendix 11a). One study included only hypertensive patients [14], while two [12, 18] excluded patients with premorbid chronic hypertension. Six out of 12 studies used renal outcome as the primary endpoint (Additional file 1: Appendix 10).

**Table 1:** Characteristics of the included studies

Patient group	Study ID	Sample size	Setting of AKI	Lower BP target arm		Higher BP target arm		AKI definition
				Target	Average of intervention	Target	Average time of intervention	
Shock	Asfar et al. [9]	776	Septic shock	MAP 65-70 mmHg	5 days	MAP 80-85 mmHg	5 days	Rates of doubling of baseline creatinine level
	Grand et al. [8]	50	Out-of-hospital cardiac arrest	MAP 65 mmHg	48 h	MAP 72 mmHg	48 h	Not reported
	Lamontagne et al. [10]	2463	Vasodilatory shock	MAP 60-65 mmHg	Median (IQR): 33.0 (15.0 to 56.0) hours	Usual care MAP median (IQR): 72.6 (69.4 to 76.5)	Median (IQR): 38.0 (19.0 to 67.0) hours	Severe acute renal failure (KDIGO stage 3 criteria)
Non-cardiac surgery	Futier et al. [11]	292	Major surgery	SBP not lower than 80 mmHg or 40% of patient’s reference value (MAP = 75 ± 13 mmHg)	Median (IQR) 465 (390–600) min	SBP remained within ± 10% of the reference value (MAP = 81 ± 14 mmHg)	Median (IQR) 423 (342–550) min	RIFLE
	Hu et al. [12]	298	Non-cardiothoracic surgery	MAP 60-70 mmHg	Median (IQR) 228 (189–252) min	MAP 95-100 mmHg	Median (IQR) 211 (188–251) min	KDIGO creatinine-based criteria
	Wanner et al. [13]	451	Major noncardiac surgery	MAP ≥ 60 mm Hg	Median (IQR) 5.4 (4.3–7.0) hours	MAP ≥ 75 mm Hg	Median (IQR) 5.3 (4.2–7.1) hours	AKI AKIN criteria
	Wu et al. [14]	646	Elective major gastrointestinal surgery	Level I MAP 65-79 mmHg	220.6 ± 71.0 min	Level II MAP 80-95 mmHg and level III MAP 96-110 mmHg	Level II 212.9 ± 73.6 and level III 218.9 ± 69.2 min	KDIGO any stages
Cardiac surgery	Azau et al. [15]	292	Elective cardiac surgery	MAP 50-60 mmHg	113 ± 51 min	MAP 75-85 mmHg	118 ± 43 min	RIFLE
	Kandler et al. [16]	90	Cardiopulmonary bypass (CABG)	Standard care (47 ± 5 mmHg)	130 ± 36 min	High arterial pressure (> 60 mmHg)	130 ± 31 min	RIFLE
	Siepe et al. [17]	92	CABG	MAP 60-70 mmHg	101 + 25 min	MAP 80-90 mmHg	91 + 30 min	Unknown
	Sirvinska et al. [19]	122	CABG surgery on CPB	MAP < 60 mmHg	90.1 ± 28.9	MAP 60-70 mmHg MAP > 70mmHG	109.5 ± 43.7 109.7 ± 45.3	RIFLE
	Vedel et al. [18]	197	elective or subacute on-pump coronary artery bypass grafting and/or left-sided heart	MAP 40-50 mmHg	94.0 ± 33.0 min	MAP 70-80 mmHg	105.6 ± 77.4 min	Rates of doubling of baseline creatinine level

Baseline characteristics of patients varied across studies in terms of age and sex. Most studies recruited elderly participants. Male sex ranged from 40 to 94% across all arms of the included studies. A total of 10 out of 12 studies reported baseline renal functions (Additional file 1: Appendix 12).

We found varied BP targets of intervention across the studies. All studies used mean arterial blood pressure (MAP) as targets for comparison, except for Futier et al. [11] which used systolic blood pressure (SBP). The lowest MAP target was 40 mmHg in Vedel et al. 2018 [18], while the highest was 110 mmHg in Wu et al. 2017 [14]. Two studies compared MAP targets with usual or standard care [10, 16]. One study compared BP targets in an individualized manner [11]. All included studies used a two-armed design, except for Sirvinskas et al. [19] and Wu et al. [14] which had three MAP target arms. The duration of intervention of shock, cardiac and non-cardiac surgery patients ranged from 1–5 days, 3–7 h and 1,5–2 h, respectively (Additional file 1: Appendix 11b).

### Risk of bias and level of certainty assessment

Nine out of the 12 studies had an overall low RoB (Additional file 1: Appendix 13). We encountered high RoB or some concerns in three domains including “Deviations from the intended interventions,” “Measurement of the outcome” and “Selection of the reported result”. All the studies had low RoB in domains “Missing outcome data” and “Measurement of the outcome.” Across 9 main comparisons, we assessed the certainty of the body of evidence as “very low” in two findings (rates of AKI and rates of RRT receipt on cardiac surgery patients) due to RoB and imprecision, 4 findings in shock patients have moderate to high level of certainty (Additional file 1: Appendix 14). Slight asymmetry funnel plot might suggest publication bias in cardiac and non-cardiac surgery studies (Additional file 1: Figure S1).

### Effect of higher MAP on AKI in shock patients.

**Fig. 2:**
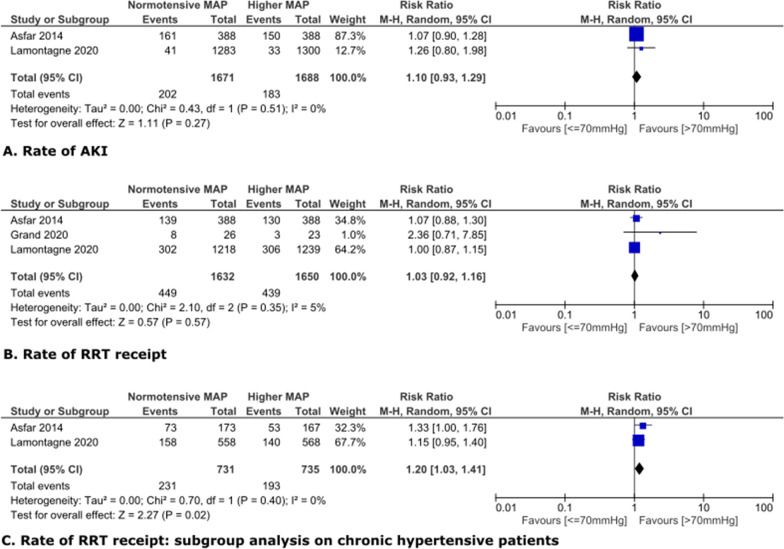
Higher MAP versus normotension in shock patients

In shock patients, the common normotensive range was 65-70 mmHg (Fig. 2). Targeting MAP higher than normotension did not significantly prevent AKI progression or reduce RRT receipt rate, with risk ratios and 95% CIs of 1.10 [0.93, 1.29] and 1.03 [0.92, 1.16], respectively (Fig. 2A,B). However, subgroup analysis on 1466 shock patients with premorbid hypertension revealed significantly lower risk of RRT receipt in higher MAP arm, with RR and 95% CI being 1.20 [1.03, 1.41] and p < 0.05 (Fig. 2C). All three comparisons had low heterogeneity. Subgroup analysis on 1767 shock patients without premorbid hypertension showed no significant difference (Additional file 1: Figure S2).

### Effect of higher MAP on AKI in cardiac surgery patients.

**Fig. 3:**
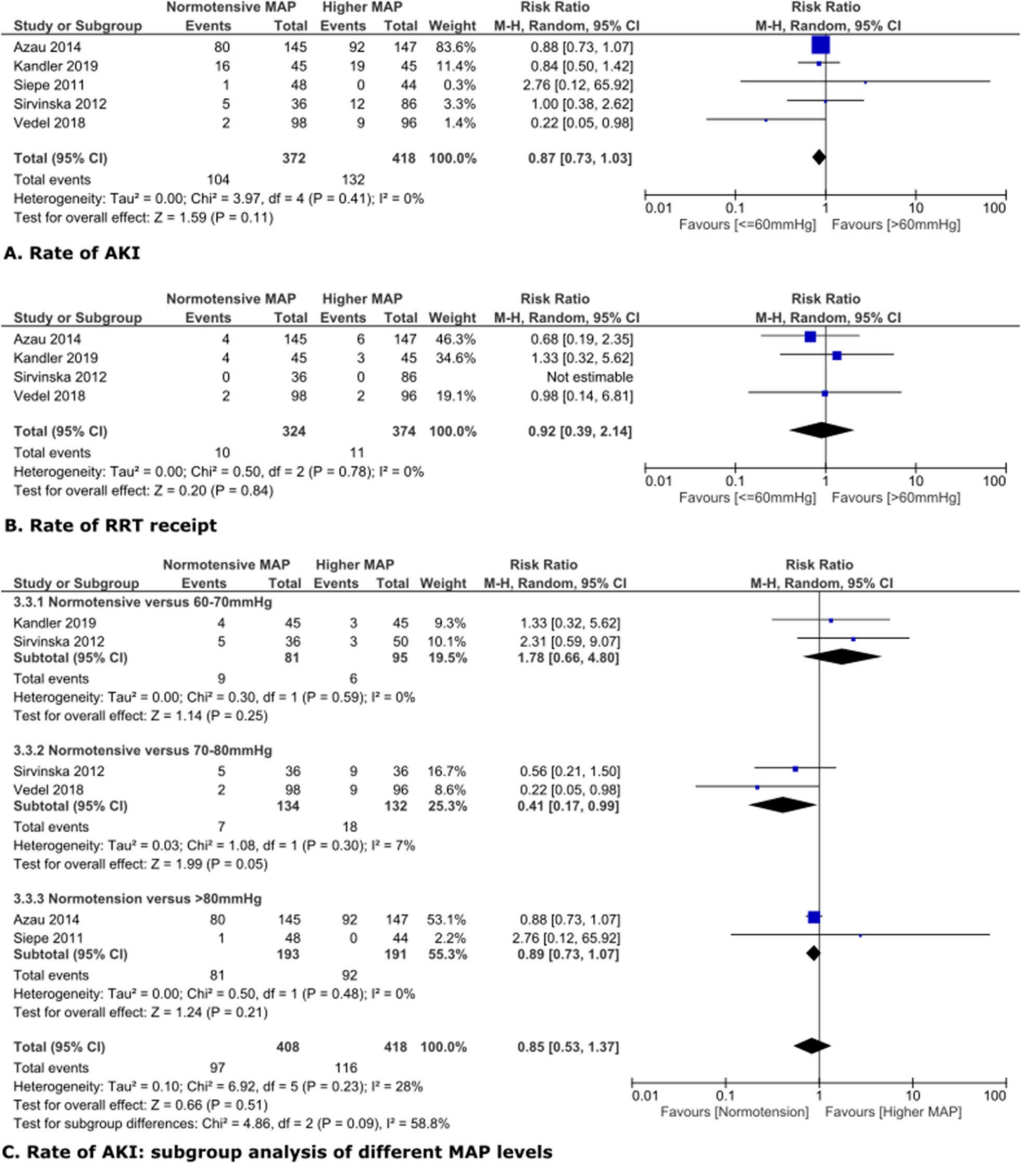
Higher MAP versus normotension in cardiac surgery patients

In cardiac surgery patients, the common normotensive level was around 40-60 mmHg. Targeting MAP above 60 mmHg did not result in significantly lower rate of AKI or RRT receipt, with risk RRs and 95% CIs of 0.87 [0.73, 1.03], 0.92 [0.39, 2.14], respectively (Fig. 3A,B). Both comparisons had low heterogeneity (Fig. 3A,B). Subgroup analysis on different levels of high MAP (60-70 mmHg, 70-80 mmHg and above 80 mmHg) versus normotension on AKI prevention did not show significant differences, with RR and 95%CI at 1.78 [0.94, 2.17], 0.41 [0.17, 0.99] and 0.89 [0.73, 1.07], respectively, all with p-value at least 0.05 (Fig. 3C).

### Effect of higher MAP on AKI in non-cardiac surgery patients

**Fig. 4:**
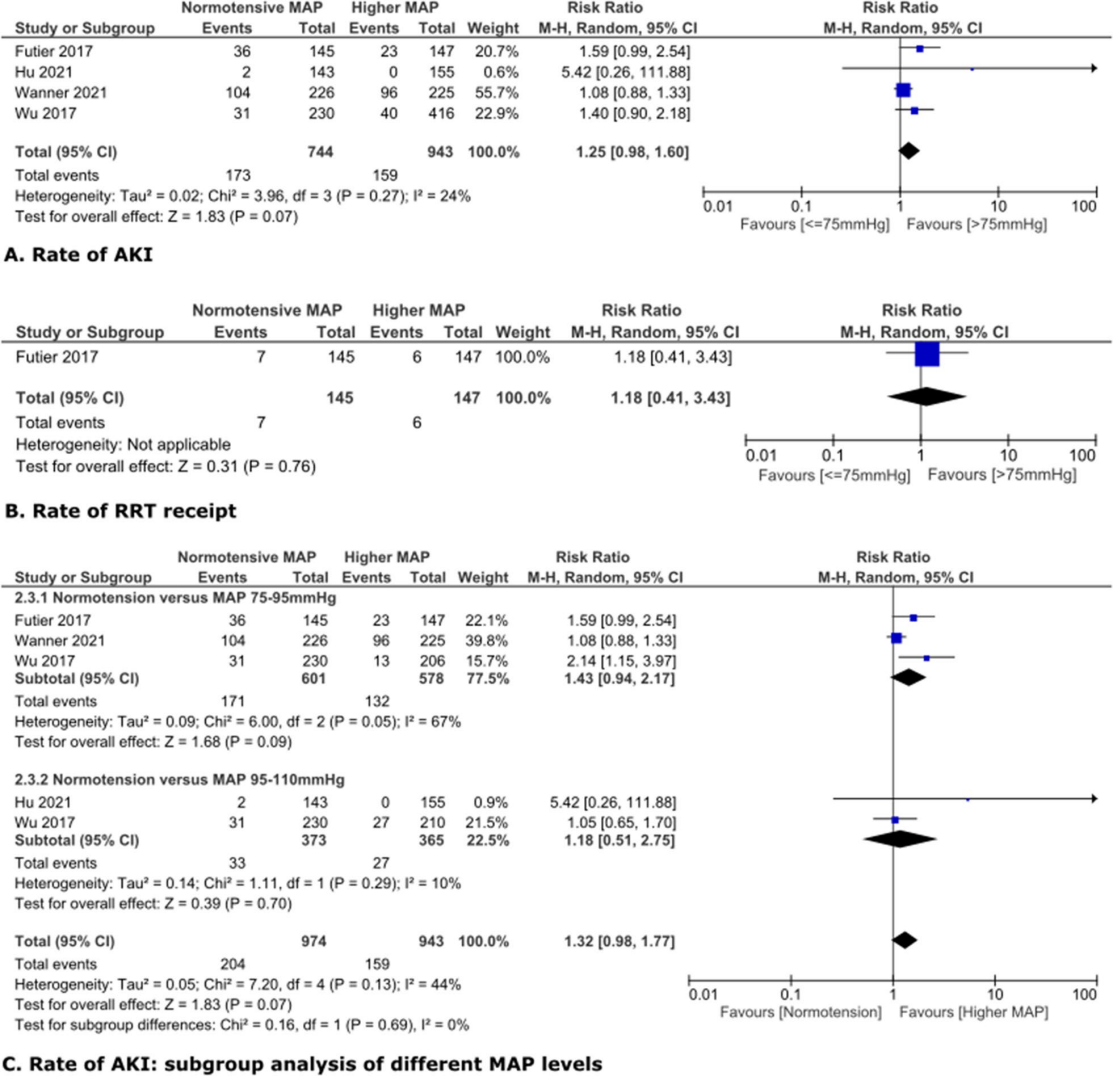
Higher MAP versus normotension in non-cardiac surgery patients

In non-cardiac surgery patients, the common normotensive range was 60–75 mmHg. Targeting higher MAP as compared to normotension did not result in significantly lower AKI or RRT receipt rates, with risk RRs and 95% CIs being 1.25 [0.98, 1.60] and 1.18 [0.41, 3.43], respectively (Fig. 4A,B). Subgroup analysis on different levels of high MAP (75–95 mmHg and 95–110 mmHg) versus normotension did not show significantly different AKI rate, with RR and 95%CI at 1.43 [0.94, 2.17] and 1.18 [0.51, 2.75], respectively (Fig. 4C).

### Meta-regression models to study the effect modification of RoB, age and hypertension on MAP-AKI relationship.

**Fig. 5:**
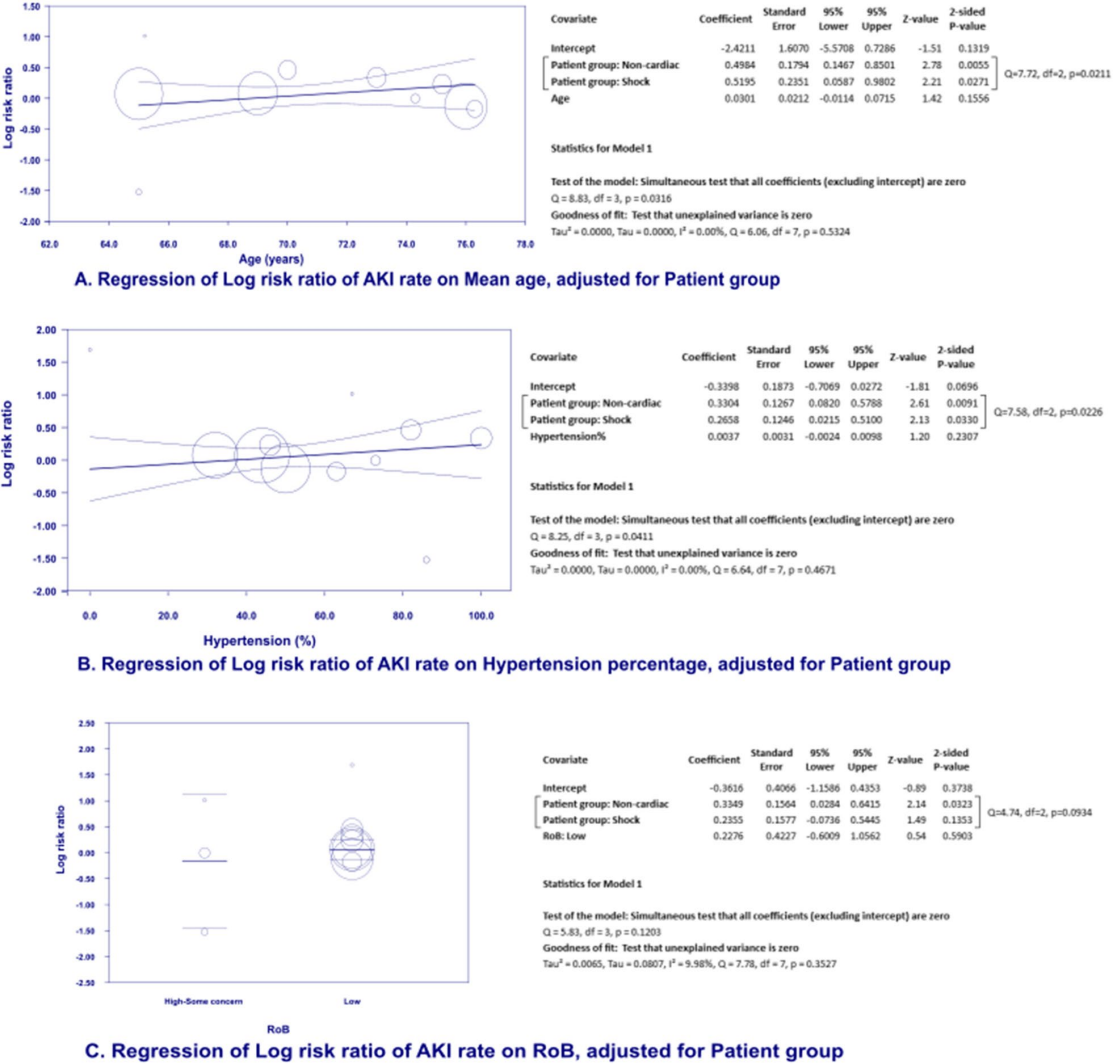
Regression of log risk ratio of AKI rate on age, hypertension percentage and RoB

Adjusting for patient group, increase in each percentage of hypertension patients in the initial cohort might lead to 0.0037 unit increase in the log RR of having AKI in normotensive MAP arms (i.e., the more hypertensive patients there are in the cohort, the lower post-intervention AKI rate in higher MAP arms). However, this effect was not significant with 95% CI at − 0.0024 to 0.0098, *p* > 0.05 (Fig. 5B). Similarly, no significant effect was observed for RoB and mean age, with the coefficients and 95% CIs of 0.23 (− 0.60, 1.06) and 0.03 (− 0.01, 0.07), respectively (Fig. 5A–C). Meta-regression models of these moderators on the association of MAP-RRT receipt rate also found no significant effects (Additional file 1: Figure S3–S5).

## Discussion

In this systematic review with meta-analysis, we found that in shock and perioperative patients, targeting higher MAP generally was not superior to normotension in terms of preventing AKI occurrence or progression. In shock patients, our study revealed that MAP above normotension (65–70 mmHg) did not result in reduced post-intervention AKI or RRT receipt rate. A patient-level pooled analysis also demonstrated that higher MAP had no effect on 28-day death or persistent organ dysfunction rate [20]. Since higher MAP might not be beneficial in AKI prevention and might even be associated with undesirable effects, a normotensive MAP should be more appropriate in this population. In cardiac surgery patients, a meta-analysis on 8 RCTs in on-pump cardiac surgery patients found no difference in rate of AKI or mortality between the different MAP arms [21]. Five out of these 8 RCTs were included in our work and yielded similar findings. Subgroup analyses on different MAP levels compared to normotension demonstrated that the wider distance between high and normotension MAP did not improve AKI prevention either.

In non-cardiac surgery patients, our study found no superiority of higher MAP in AKI prevention, as compared to normotension. One meta-analysis investigated the effects of strict intraoperative BP management strategy (defined as MAP ≥ 70 mmHg/MAP decrease less than 30% from the baseline) on postoperative AKI [22]. The authors found that strict BP management might significantly reduce the incidence of post-operative AKI, with RR [CI95%] of 0.73 [0.58–0.92] [22]. The different results might be due to the included RCTs. We selected RCTs that used BP as the only target, while three out of five RCTs in previous study [22] used a multi-modality strategy. For example, the RCT by Schmid S. et al. used a goal-directed hemodynamic management, optimized pain therapy, oxygen therapy, and optimized nutrition in the intervention group [23]. Therefore, the role of higher MAP in postoperative AKI prevention might be questioned. Our current work did not suggest the use of high MAP to prevent post-operative AKI.

MAP is the fundamental driver of organ perfusion. Autoregulation is the capacity of healthy essential organs such as the heart, brain, and kidney to maintain a consistent blood flow rate within a range of perfusion pressure [24]. In disrupted hemodynamics, such as circulatory shock and perioperative patients, organ blood flow depends on perfusion pressure. Targeting a physiological MAP is necessary to safeguard key organs by ensuring peripheral perfusion BP and microcirculatory blood flow [24]. As a target for hemodynamic optimization, MAP is commonly set at 65 mmHg in septic shock guidelines [25] and perioperative settings [26]. However, since the kidney has the highest autoregulation threshold compared to other organs [27], it remains unclear if a higher MAP is required for better AKI prevention. Animal research demonstrated a 50 to 90 mmHg renal autoregulation threshold, but no human data was available [28]. The absence of renoprotective impact of greater MAP relative to normotension may be related to the kidney's ability to autoregulate above the autoregulation threshold. Our findings supported the currently suggested normotension level (MAP about 65 mmHg) over higher MAP targets for AKI prevention in frequent AKI-related scenarios.

After adjusting for patient group, we found no significant effect of hypertension, mean age, or RoB on the link between MAP and AKI in meta-regression models. Nevertheless, subgroup analysis of shock patients with hypertension revealed a renoprotective signal of higher MAP (> 70 mmHg) on reducing the RRT receipt rate. Chronic hypertension is known to shift the renal autoregulation zone to the right, higher MAP is therefore required to maintain adequate perfusion pressure [29]. Dewitte et al. studied 26 hypertensive patients with sepsis-associated AKI and discovered that targeting 80-85 mmHg MAP was linked with significantly higher creatinine clearance than lower MAP (65-70 mmHg) [30]. Evidence of effect modification of hypertension is scarce in cardiac and non-cardiac surgery patients. Wu et al.'s RCT, which included exclusively hypertensive patients undergoing major surgery, demonstrated renoprotective effect of MAP at 80–95 mmHg versus MAP of 65–79 mmHg. Guidelines in resuscitating shock patients [31], consensus on post-operative non-cardiac AKI [32] and perioperative patients [33] also recommended higher MAP target in hypertension patients. Due to the observational nature of subgroup analysis and meta-regression, whether higher MAP targets could actually prevent AKI in hypertensive patients with hemodynamic instability may be an essential question for future trials.

This study has several strengths including a pre-registered protocol, a comprehensive literature search and an updated RoB version. Exclusive inclusion of RCTs comparing different MAP targets on renal outcome helps to isolate the effect of MAP and vasopressor use on AKI prevention and management, whereas extensive selection of different AKI-related settings broadens the scope of the review. Major limitation of this review was the limited number of studies in each patient group, which might link to underpowered statistical findings. This also rendered the multivariable analysis in the meta-regression models. Due to lack of reported results, many subgroup analyses could not be performed. Second, included RCTs had varied AKI definitions, and most did not employ kidney function as a primary objective. Sample sizes therefore may not be powered to detect difference in kidney outcomes. Lastly, some results (hypertension percentage) were not reported in the original publications, and we imputed them using the percentage of anti-hypertension medications, which could be a close approximation.

## Conclusions

Targeting a higher MAP in shock or perioperative patients may not be superior to normotension in terms of reducing the onset or progression of AKI. Targeting MAP over 70 mmHg in shock patients with premorbid hypertension may reduce the RRT administration rate, suggesting a renoprotective impact. Considering the limitations of the present evidence, additional studies are required to assess the benefits of a high MAP target in preventing AKI in hypertensive patients in common settings of hemodynamic instability.

## Supplementary Information

**Additional file 1.** Supplemental digital content.

## Data Availability

The datasets used and analyzed during the current study are available from the corresponding author on reasonable request.

## Abbreviations

AKI: Acute kidney injury
BP: Blood pressure
MAP: Mean arterial pressure
RCT: Randomized controlled trial
RoB: Risk of bias
RR: Risk ratio
RRT: Renal replacement therapy
SBP: Systolic blood pressure

## Code availability

Code is available in the electronic supplementary materials.
