# Supplementary material for: Higher blood pressure versus normotension targets to prevent acute kidney injury: a systematic review and meta-regression of randomized controlled trials
Source: Crit Care. 2022 Nov 25;26:364. doi: 10.1186/s13054-022-04236-1 (PMC9700976; doi:10.1186/s13054-022-04236-1)
Supplement: Supplementary file 1 — Additional file 1. Supplemental digital content. [file 13054_2022_4236_MOESM1_ESM.docx]

**SUPPLEMENTAL DIGITAL CONTENT**

**Supplement to: Higher blood pressure versus normotension targets to prevent acute kidney injury:**

**A systematic review and meta-regression of randomized controlled trials.**

**This supplemental digital content was submitted to Critical Care**

**Nguyen Trong Phu Tran^12*^, Prit Kusirisin^1234^, Piyanut Kaewdoungtien^1236^, Jeerath Phannajit^15^, Nattachai Srisawat^1237^**

**Author affiliations:**

1. Division of Nephrology, Department of Medicine, Faculty of Medicine, Chulalongkorn University, Bangkok, Thailand.

2. Excellence Center for Critical Care Nephrology, King Chulalongkorn Memorial Hospital, Bangkok, Thailand.

3. Center of Excellence in Critical Care Nephrology, Faculty of Medicine, Chulalongkorn University, Bangkok, Thailand

4. Division of Nephrology, Department of Internal Medicine, Faculty of Medicine, Chiang Mai University, Chiang Mai, Thailand.

5. Division of Clinical Epidemiology, Department of Medicine, Faculty of Medicine, Chulalongkorn University

6. Division of Nephrology, Police General Hospital, Royal Thai Police Headquarters, Bangkok, Thailand.

7. Academy of Science, Royal Society of Thailand, Bangkok, Thailand.

***Corresponding author:**

Nattachai Srisawat, MD, PhD

Division of Nephrology, Department of Medicine, Faculty of Medicine, King Chulalongkorn Memorial Hospital, Bangkok 10330, Thailand.

Phone: (662)-2564000 Ext. 3597

Email: drnattachai@yahoo.com

**TABLE OF CONTENTS**

[Appendix 1. Ovid MEDLINE search queries 4](#_Toc107399940)

[Appendix 2. EMBASE search queries 8](#_Toc107399941)

[Appendix 3. SCOPUS search queries (Date: 13/05/2022) 10](#_Toc107399942)

[Appendix 4. COCHRANE LIBRARY search queries 14](#_Toc107399943)

[Appendix 5. CLINICALTRIALS.gov Search Strategy 16](#_Toc107399944)

[Appendix 6. WHO International Clinical Trials Registry Platform (ICTRP) Search Strategy 17](#_Toc107399945)

[Appendix 7. SUMMARY OF IMPORTANT EXCLUDED STUDIES WITH REASONS 18](#_Toc107399946)

[Appendix 8. SUMMARY OF STUDIES AWAITING CLASSIFICATION 23](#_Toc107399947)

[Appendix 9. SUMMARY OF ONGOING RANDOMIZED CLINICAL TRIALS 25](#_Toc107399948)

[Appendix 10. SUMMARY OF OUTCOMES REPORTED IN THE INCLUDED STUDIES AND DEFINITIONS USED FOR AKI 29](#_Toc107399949)

[Appendix 11a. SUMMARY OF CHARACTERISTICS OF THE INCLUDED STUDIES 33](#_Toc107399950)

[Appendix 11b. SUMMARY OF CHARACTERISTICS OF THE INCLUDED STUDIES (CONTINUED) 38](#_Toc107399951)

[Appendix 12. AVAILABLE BASELINE CHARACTERISTICS OF PATIENTS IN THE INCLUDED STUDIES 41](#_Toc107399952)

[Appendix 13. RISK OF BIAS ASSESSMENT OF THE INCLUDED STUDIES 42](#_Toc107399953)

[Appendix 14. SUMMARY OF FINDINGS AND LEVEL OF CERTAINTY ASSESSMENT OF THE BODY OF EVIDENCE 43](#_Toc107399954)

[Appendix 15. S-Figure 1. FUNNEL PLOT OF INCLUDED STUDY WITH OUTCOME OF AKI RATE 46](#_Toc107399955)

[Appendix 16. S-Figure 2. Higher MAP versus normotension in shock patients without hypertension 47](#_Toc107399956)

[Appendix 17. S-Figure 3. Meta-regression of Log risk ratio of RRT rate on Age 48](#_Toc107399957)

[Appendix 18. S-Figure 4. Meta-regression of Log risk ratio of RRT rate on Hypertension percentage and RoB 49](#_Toc107399958)

[REFERENCES 51](#_Toc107399959)

# Appendix 1. Ovid MEDLINE search queries

Ovid MEDLINE(R) ALL <1946 to May 11, 2022>

1 exp "Critical Care"/ 64193

2 exp "Intensive Care Units"/ 99736

3 "intensive care".tw. 170858

4 exp "Critical Illness"/ 35880

5 "critical care".tw. 32243

6 ICU.tw. 72313

7 "critically ill".tw. 53901

8 "critical illness".tw. 10308

9 shock.mp. or exp Shock/ 263361

10 exp Hypotension/ 29284

11 hypotens*.mp. 86938

12 exp Sepsis/ or sepsis.mp. 199919

13 cardiac arrest.mp. or exp Heart Arrest/ 70299

14 surgery.mp. or exp General Surgery/ 2904101

15 exp Postoperative Complications/ 593362

16 1 or 2 or 3 or 4 or 5 or 6 or 7 or 8 or 9 or 10 or 11 or 12 or 13 or 14 or 15 3776433

17 RCT.mp. 29690

18 rct*.mp. 65236

19 randomized controlled trial.mp. or exp Randomized Controlled Trial/ 606337

20 (randomized adj3 control : adj3 trial :).mp. 958956

21 exp Controlled Clinical Trial/ 659589

22 (controlled adj3 clinical adj3 trial).mp. 116453

23 (clinical adj3 trial :).mp. 4739343

24 (experimental adj3 trial :).mp. 1463317

25 exp Clinical Study/ 1081506

26 (clinical adj3 study).mp. 142902

27 Comparative Study/ 1911052

28 (comparative adj3 study).mp. 1962461

29 exp Evaluation Studies/ 261701

30 (evaluation adj3 study).mp. 279875

31 exp Multicenter Study/ 321159

32 (multicenter adj3 study).mp. 338028

33 17 or 18 or 19 or 20 or 21 or 22 or 23 or 24 or 25 or 26 or 27 or 28 or 29 or 30 or 31 or 32 8128051

34 16 and 33 1291955

35 exp Arterial Pressure/ 6716

36 MAP.mp. 232020

37 (mean arterial adj2 pressure).mp. 44249

38 mean arterial pressure.mp. 33916

39 mean arterial blood pressure.mp. 10815

40 35 or 36 or 37 or 38 or 39 265721

41 (target* or level* or group* or optim*).mp. 9752562

42 (mmHg or mm Hg).mp. 161973

43 40 and 41 and 42 10768

44 34 and 43 2837

45 limit 44 to humans 1642

# Appendix 2. EMBASE search queries

Search Date: 12/5/2022

| **No.** | **Query results** | **Results** |
| --- | --- | --- |
| #48 | #46 NOT #47 | 4991 |
| #47 | #44 AND ([adult]/lim OR [aged]/lim OR [middle aged]/lim OR [very elderly]/lim OR [young adult]/lim) AND ('Article'/it OR 'Article in Press'/it OR 'Conference Abstract'/it OR 'Conference Paper'/it) AND 'retrospective study'/de | 886 |
| #46 | #44 AND ([adult]/lim OR [aged]/lim OR [middle aged]/lim OR [very elderly]/lim OR [young adult]/lim) AND ('Article'/it OR 'Article in Press'/it OR 'Conference Abstract'/it OR 'Conference Paper'/it) | 5704 |
| #45 | #44 AND ([adult]/lim OR [aged]/lim OR [middle aged]/lim OR [very elderly]/lim OR [young adult]/lim) | 5752 |
| #44 | #33 AND #42 AND [humans]/lim AND [clinical study]/lim | 7268 |
| #43 | #33 AND #42 | 8056 |
| #42 | #39 AND #40 AND #41 | 29458 |
| #41 | mmhg OR 'mm hg' | 247032 |
| #40 | target* OR level* OR group* OR optim* | 14402341 |
| #39 | #34 OR #35 OR #36 OR #37 OR #38 | 366428 |
| #38 | 'mean arterial blood pressure' | 13244 |
| #37 | 'mean arterial pressure' | 80512 |
| #36 | 'mean arterial' NEAR/2 pressure | 90414 |
| #35 | map | 235840 |
| #34 | 'arterial pressure'/exp | 134753 |
| #33 | #16 AND #32 | 3932237 |
| #32 | #17 OR #18 OR #19 OR #20 OR #21 OR #22 OR #23 OR #24 OR #25 OR #26 OR #27 OR #28 OR #29 OR #30 OR #31 | 12327351 |
| #31 | 'comparative study'/de | 950450 |
| #30 | multicenter NEAR/3 study | 377788 |
| #29 | 'multicenter study'/exp | 306356 |
| #28 | evaluation NEAR/3 study | 244400 |
| #27 | 'evaluation studies'/exp | 245807 |
| #26 | comparative NEAR/3 study | 1024251 |
| #25 | clinical NEAR/3 study | 4614616 |
| #24 | 'clinical study'/exp | 11007800 |
| #23 | experimental NEAR/3 'trial :' | 3357 |
| #22 | clinical NEAR/3 'trial :' | 1691859 |
| #21 | controlled NEAR/3 clinical NEAR/3 trial | 467007 |
| #20 | 'controlled clinical trial'/exp | 863679 |
| #19 | randomized NEAR/3 'control :' NEAR/3 'trial :' | 9579 |
| #18 | 'randomized controlled trial' OR 'randomized controlled trial'/exp | 917453 |
| #17 | rct | 50050 |
| #16 | #1 OR #2 OR #3 OR #4 OR #5 OR #6 OR #7 OR #8 OR #9 OR #10 OR #11 OR #12 OR #13 OR #14 OR #15 | 7178532 |
| #15 | 'postoperative complications'/exp | 782866 |
| #14 | surgery OR 'general surgery'/exp | 5474806 |
| #13 | 'cardiac arrest' OR 'heart arrest'/exp | 122955 |
| #12 | 'sepsis'/exp OR sepsis | 345735 |
| #11 | hypotens* | 198796 |
| #10 | 'hypotension'/exp | 161704 |
| #9 | shock OR 'shock'/exp | 390193 |
| #8 | 'critical illness':ti,ab | 14847 |
| #7 | 'critically ill':ti,ab | 76672 |
| #6 | icu:ti,ab | 138009 |
| #5 | 'critical care':ti,ab | 53249 |
| #4 | 'critical illness'/exp | 32287 |
| #3 | 'intensive care':ti,ab | 240341 |
| #2 | 'intensive care unit'/exp | 235826 |
| #1 | 'critical care'/exp OR 'critical care' | 1089196 |

# Appendix 3. SCOPUS search queries (Date: 13/05/2022)

| Step | Search string | Results |
| --- | --- | --- |
| 7 | ( ( ( INDEXTERMS ( "Critical Care" ) OR INDEXTERMS ( "Intensive Care Units" ) OR TITLE-ABS ( "intensive care" ) OR INDEXTERMS ( "Critical Illness" ) OR TITLE-ABS ( "critical care" ) OR TITLE-ABS ( icu ) OR TITLE-ABS ( "critically ill" ) OR TITLE-ABS ( "critical illness" ) ) OR ( TITLE-ABS-KEY ( shock ) OR INDEXTERMS ( shock ) OR INDEXTERMS ( hypotension ) OR TITLE-ABS-KEY ( hypotens* ) INDEXTERMS ( sepsis ) OR TITLE-ABS-KEY ( sepsis ) OR TITLE-ABS-KEY ( "cardiac arrest" ) OR INDEXTERMS ( "Heart Arrest" ) OR TITLE-ABS-KEY ( surgery ) OR INDEXTERMS ( "General Surgery" ) OR INDEXTERMS ( "Postoperative Complications" ) ) ) AND ( TITLE-ABS-KEY ( rct ) OR TITLE-ABS-KEY ( rct* ) OR TITLE-ABS-KEY ( "randomized controlled trial" ) OR INDEXTERMS ( "Randomized Controlled Trial" ) OR TITLE-ABS-KEY ( randomized W/3 "control :" W/3 "trial :" ) OR INDEXTERMS ( "Controlled Clinical Trial" ) OR TITLE-ABS-KEY ( controlled W/3 clinical W/3 trial ) TITLE-ABS-KEY ( clinical W/3 "trial :" ) OR TITLE-ABS-KEY ( experimental W/3 "trial :" ) OR INDEXTERMS ( "Clinical Study" ) OR TITLE-ABS-KEY ( clinical W/3 study ) OR INDEXTERMS ( "Comparative Study" ) OR TITLE-ABS-KEY ( comparative W/3 study ) OR INDEXTERMS ( "Evaluation Studies" ) OR TITLE-ABS-KEY ( evaluation W/3 study ) OR INDEXTERMS ( "Multicenter Study" ) OR TITLE-ABS-KEY ( multicenter W/3 study ) ) ) AND ( ( INDEXTERMS ( "Arterial Pressure" ) OR TITLE-ABS-KEY ( map ) OR TITLE-ABS-KEY ( "mean arterial" W/2 pressure ) OR TITLE-ABS-KEY ( "mean arterial pressure" ) OR TITLE-ABS-KEY ( "mean arterial blood pressure" ) ) AND ( TITLE-ABS-KEY ( target* OR level* OR group* OR optim* ) ) AND ( TITLE-ABS-KEY ( mmhg OR "mm Hg" ) ) ) | 465 document results |
| 6 | ( INDEXTERMS ( "Arterial Pressure" ) OR TITLE-ABS-KEY ( map ) OR TITLE-ABS-KEY ( "mean arterial" W/2 pressure ) OR TITLE-ABS-KEY ( "mean arterial pressure" ) OR TITLE-ABS-KEY ( "mean arterial blood pressure" ) ) AND ( TITLE-ABS-KEY ( target* OR level* OR group* OR optim* ) ) AND ( TITLE-ABS-KEY ( mmhg OR "mm Hg" ) ) | 16,647 document results |
| 5 | ( ( INDEXTERMS ( "Critical Care" ) OR INDEXTERMS ( "Intensive Care Units" ) OR TITLE-ABS ( "intensive care" ) OR INDEXTERMS ( "Critical Illness" ) OR TITLE-ABS ( "critical care" ) OR TITLE-ABS ( icu ) OR TITLE-ABS ( "critically ill" ) OR TITLE-ABS ( "critical illness" ) ) OR ( TITLE-ABS-KEY ( shock ) OR INDEXTERMS ( shock ) OR INDEXTERMS ( hypotension ) OR TITLE-ABS-KEY ( hypotens* ) INDEXTERMS ( sepsis ) OR TITLE-ABS-KEY ( sepsis ) OR TITLE-ABS-KEY ( "cardiac arrest" ) OR INDEXTERMS ( "Heart Arrest" ) OR TITLE-ABS-KEY ( surgery ) OR INDEXTERMS ( "General Surgery" ) OR INDEXTERMS ( "Postoperative Complications" ) ) ) AND ( TITLE-ABS-KEY ( rct ) OR TITLE-ABS-KEY ( rct* ) OR TITLE-ABS-KEY ( "randomized controlled trial" ) OR INDEXTERMS ( "Randomized Controlled Trial" ) OR TITLE-ABS-KEY ( randomized W/3 "control :" W/3 "trial :" ) OR INDEXTERMS ( "Controlled Clinical Trial" ) OR TITLE-ABS-KEY ( controlled W/3 clinical W/3 trial ) TITLE-ABS-KEY ( clinical W/3 "trial :" ) OR TITLE-ABS-KEY ( experimental W/3 "trial :" ) OR INDEXTERMS ( "Clinical Study" ) OR TITLE-ABS-KEY ( clinical W/3 study ) OR INDEXTERMS ( "Comparative Study" ) OR TITLE-ABS-KEY ( comparative W/3 study ) OR INDEXTERMS ( "Evaluation Studies" ) OR TITLE-ABS-KEY ( evaluation W/3 study ) OR INDEXTERMS ( "Multicenter Study" ) OR TITLE-ABS-KEY ( multicenter W/3 study ) ) | 25,847document results |
| 4 | TITLE-ABS-KEY ( rct ) OR TITLE-ABS-KEY ( rct* ) OR TITLE-ABS-KEY ( "randomized controlled trial" ) OR INDEXTERMS ( "Randomized Controlled Trial" ) OR TITLE-ABS-KEY ( randomized W/3 "control :" W/3 "trial :" ) OR INDEXTERMS ( "Controlled Clinical Trial" ) OR TITLE-ABS-KEY ( controlled W/3 clinical W/3 trial ) TITLE-ABS-KEY ( clinical W/3 "trial :" ) OR TITLE-ABS-KEY ( experimental W/3 "trial :" ) OR INDEXTERMS ( "Clinical Study" ) OR TITLE-ABS-KEY ( clinical W/3 study ) OR INDEXTERMS ( "Comparative Study" ) OR TITLE-ABS-KEY ( comparative W/3 study ) OR INDEXTERMS ( "Evaluation Studies" ) OR TITLE-ABS-KEY ( evaluation W/3 study ) OR INDEXTERMS ( "Multicenter Study" ) OR TITLE-ABS-KEY ( multicenter W/3 study ) | 825,971 document results |
| 3 | ( INDEXTERMS ( "Critical Care" ) OR INDEXTERMS ( "Intensive Care Units" ) OR TITLE-ABS ( "intensive care" ) OR INDEXTERMS ( "Critical Illness" ) OR TITLE-ABS ( "critical care" ) OR TITLE-ABS ( icu ) OR TITLE-ABS ( "critically ill" ) OR TITLE-ABS ( "critical illness" ) ) OR ( TITLE-ABS-KEY ( shock ) OR INDEXTERMS ( shock ) OR INDEXTERMS ( hypotension ) OR TITLE-ABS-KEY ( hypotens* ) INDEXTERMS ( sepsis ) OR TITLE-ABS-KEY ( sepsis ) OR TITLE-ABS-KEY ( "cardiac arrest" ) OR INDEXTERMS ( "Heart Arrest" ) OR TITLE-ABS-KEY ( surgery ) OR INDEXTERMS ( "General Surgery" ) OR INDEXTERMS ( "Postoperative Complications" ) ) | 466,735 document results |
| 2 | TITLE-ABS-KEY ( shock ) OR INDEXTERMS ( shock ) OR INDEXTERMS ( hypotension ) OR TITLE-ABS-KEY ( hypotens* ) INDEXTERMS ( sepsis ) OR TITLE-ABS-KEY ( sepsis ) OR TITLE-ABS-KEY ( "cardiac arrest" ) OR INDEXTERMS ( "Heart Arrest" ) OR TITLE-ABS-KEY ( surgery ) OR INDEXTERMS ( "General Surgery" ) OR INDEXTERMS ( "Postoperative Complications" ) | 103,797 document results |
| 1 | INDEXTERMS ( "Critical Care" ) OR INDEXTERMS ( "Intensive Care Units" ) OR TITLE-ABS ( "intensive care" ) OR INDEXTERMS ( "Critical Illness" ) OR TITLE-ABS ( "critical care" ) OR TITLE-ABS ( icu ) OR TITLE-ABS ( "critically ill" ) OR TITLE-ABS ( "critical illness" ) | 383,128 document results |

# Appendix 4. COCHRANE LIBRARY search queries

(Last search 13/5/2022)

ID Search (Hits)

#1 [mh "Critical Care"] (2163)

#2 [mh "Intensive Care Units"] (3887)

#3 "intensive care":ti,ab (20876)

#4 [mh "Critical Illness"] (2483)

#5 "critical care":ti,ab (2509)

#6 ICU:ti,ab (14444)

#7 "critically ill":ti,ab (7206)

#8 "critical illness":ti,ab (1281)

#9 shock:ti,ab,kw OR [mh Shock] (12202)

#10 [mh Hypotension] (2311)

#11 hypotens*:ti,ab,kw (18671)

#12 [mh Sepsis] OR sepsis:ti,ab,kw (13977)

#13 "cardiac arrest":ti,ab,kw OR [mh "Heart Arrest"] (4775)

#14 surgery:ti,ab,kw OR [mh "General Surgery"] (228770)

#15 [mh "Postoperative Complications"] (42141)

#16 #1 OR #2 OR #3 OR #4 OR #5 OR #6 OR #7 OR #8 OR #9 OR #10 OR #11 OR #12 OR #13 OR #14 OR #15 (295701)

#17 RCT:ti,ab,kw (31408)

#18 rct*:ti,ab,kw (37400)

#19 "randomized controlled trial":ti,ab,kw OR [mh "Randomized Controlled Trial"] (550082)

#20 (randomized:ti,ab,kw NEAR/3 "control":ti,ab,kw NEAR/3 "trial":ti,ab,kw) (15928)

#21 [mh "Controlled Clinical Trial"] (128)

#22 (controlled:ti,ab,kw NEAR/3 clinical:ti,ab,kw NEAR/3 trial:ti,ab,kw) (224016)

#23 (clinical:ti,ab,kw NEAR/3 "trial":ti,ab,kw) (400680)

#24 (experimental:ti,ab,kw NEAR/3 "trial":ti,ab,kw) (2011)

#25 [mh "Clinical Study"] (145)

#26 (clinical:ti,ab,kw NEAR/3 study:ti,ab,kw) (425113)

#27 [mh ^"Comparative Study"] (5)

#28 (comparative:ti,ab,kw NEAR/3 study:ti,ab,kw) (57729)

#29 [mh "Evaluation Studies"] (1)

#30 (evaluation:ti,ab,kw NEAR/3 study:ti,ab,kw) (11636)

#31 [mh "Multicenter Study"] (10)

#32 (multicenter:ti,ab,kw NEAR/3 study:ti,ab,kw) (89331)

#33 #17 OR #18 OR #19 OR #20 OR #21 OR #22 OR #23 OR #24 OR #25 OR #26 OR #27 OR #28 OR #29 OR #30 OR #31 OR #32 (783382)

#34 #16 AND #33 (156001)

#35 [mh "Arterial Pressure"] (460)

#36 MAP:ti,ab,kw (7516)

#37 ("mean arterial":ti,ab,kw NEAR/2 pressure:ti,ab,kw) (11915)

#38 "mean arterial pressure":ti,ab,kw (10503)

#39 "mean arterial blood pressure":ti,ab,kw (2091)

#40 #35 OR #36 OR #37 OR #38 OR #39 (15909)

#41 (target*:ti,ab,kw OR level*:ti,ab,kw OR group*:ti,ab,kw OR optim*:ti,ab,kw) (1040549)

#42 (mmHg:ti,ab,kw OR "mm Hg":ti,ab,kw) (36498)

#43 #40 AND #41 AND #42 (3162)

#44 #34 AND #43 (924)

# Appendix 5. CLINICALTRIALS.gov Search Strategy

Clinical Trials.gov

Advanced search, no date limit applied

Condition or disease: Critical care OR intensive care OR critically ill OR hypoten* OR shock OR surgery OR sepsis OR perioperat* OR postoperat* OR cardiac arrest

AND

"blood pressure" OR "arterial pressure"

AND

Renal OR kidney OR urine

Applied filters: Interventional, Adult (18-64), Older Adult (65+)

137 studies

# Appendix 6. WHO International Clinical Trials Registry Platform (ICTRP) Search Strategy

Advanced search

Condition: Critical care OR intensive care OR critically ill OR hypoten* OR shock OR surgery OR sepsis OR perioperat* OR postoperat* OR cardiac arrest

AND

"blood pressure" OR "arterial pressure"

AND

Renal OR kidney OR urine

AND

Recruitment status: “all”

Phases: “all”

128 studies

# Appendix 7. SUMMARY OF IMPORTANT EXCLUDED STUDIES WITH REASONS

| **Author** | **Title** | **Participants** | **Intervention** | **Main results** | **Main reason** |
| --- | --- | --- | --- | --- | --- |
| Varajic et al 2019 (1) | High versus low mean arterial pressures in hepatorenal syndrome: a randomized controlled trial. | 18 patients with hepatorenal syndrome. | In high MAP group, vasopressor infusion was titrated to keep MAP above ≥85 mmHg.  In low MAP group, vasopressor infusion was titrated every 30 min with the goal of keeping the MAP 65–70 mmHg. | The day four urine output in the high and low MAP group was 1194  (SD = 1249) mL/24 h and 920 (SD = 812) mL/24 h, respectively (p=0.513). | Wrong population (not hypotensive patients). |
| Jozwiak 2017 (2) | Effect of two levels of mean arterial pressure on microcirculatory reserve in septic shock patients | 22 septic shock patients | MAP > 75 mmHg within the first six hours of resuscitation, then decreased to 65-70 mmHg by | The decrease in MAP was associated with a decrease in mean StO2 recovery slope (3.00 ± 1.40 vs. 2.61 ± 1.46 units/sec, respectively, p < 0.05) | -Wrong study design.  -No renal oucome data. |
| Redfors 2011 (3) | Effects of norepinephrine on renal perfusion, filtration and oxygenation in vasodilatory shock and acute kidney injury | 12 post-cardiac surgery patients with norepinephrine-dependent vasodilatory shock and AKI | NE infusion rate was randomly and sequentially titrated to target MAPs of 60, 75 and 90 mmHg | At target MAP of 75 mmHg, RDO2 (13%), GFR (27%) and urine flow were higher and renal oxygen extraction was lower (-7.4%) compared with at target MAP of 60 mmHg. However, the renal variables did not differ when compared at target MAPs of 75 and 90 mmHg | Wrong study design. |
| Shao 2019  (4) | [Effects of different target blood pressure resuscitation on peripheral blood inflammatory factors and hemodynamics in patients with traumatic hemorrhagic shock]. | 60 patients with traumatic hemorrhagic shock | According to the difference of mean arterial pressure (MAP) target, the patients were divided into low MAP (60 mmHg <= MAP < 65 mmHg, 1 mmHg = 0.133 kPa), middle MAP (65 mmHg <= MAP < 70 mmHg) and high MAP (70 mmHg <= MAP < 75 mmHg) groups | After 30 minutes of resuscitation the middle MAP group was superior to the other two groups in inhibiting the expressions of pro-inflammatory factors tumor necrosis factor-alpha (TNF-alpha), interleukin-6 (IL-6) and promoting anti-inflammatory factors IL-10 [TNF-alpha mRNA (2-DELTADELTACt) | No renal outcomes. |
| Gold 1995  (5) | Improvement of outcomes after coronary artery bypass. A randomized trial comparing intraoperative high versus low mean arterial pressure | 248 patients undergoing primary, nonemergency coronary bypass | Patients were randomized to a low mean arterial pressure of 50 to 60 mm Hg or a high mean arterial pressure of 80 to 100 mm Hg during cardiopulmonary bypass | The overall incidence of combined cardiac and neurologic complications was significantly lower in the high pressure group at 4.8% than in the low pressure group at 12.9% (p = 0.026) | No renal outcomes |
| Rose 2019  (6) | The impact of high versus low mean arterial pressure goals in cirrhotic patients with septic shock | 57 patients with cirrhosis | 28 patients randomized to the low‐target (MAP 65-70mmHg) group and 29 patients in the high‐target group (MAP 80-85mmHg) | 12/28 patients (42.8%) in the low‐target group vs. 10/29 patients (34.5%) in the high‐target group required RRT from day 0 to day 7 of treatment [OR 1.43, 95% CI (0.49‐4.16), p=0.52] | This is a subgroup analysis of patients included in the Sepsispam study by Asfar et al 2014 (7), which was already included in our meta-analysis. |
| Bootsma 2021 (8) | High Versus Normal Blood Pressure Targets in Relation to Right Ventricular Dysfunction After Cardiac Surgery: a Randomized Controlled Trial | 78 patients undergoing cardiac surgery | Normal target group (mean arterial pressure 65 mmHg) or a high target group [mean arterial pressure 85 mmHg]) | There was no significant between‐group difference in change of RVEF <20% (–1% [–3.3 to 1.8] in the normal‐target group v 0.5% [–1 to 4] in the high‐target group; p = 0.159) | No renal outcomes post-intervention. |
| Morrison 2011 (9) | Hypotensive resuscitation strategy reduces transfusion requirements and severe postoperative coagulopathy in trauma patients with hemorrhagic shock: preliminary results of a randomized controlled trial. | 90 trauma patients with hemorrhagic shock | Low mean arterial pressure arm were managed with a hypotensive resuscitation strategy in which the target MAP was 50 mm Hg. Those in the control (high MAP) arm were managed with standard fluid resuscitation to a target MAP of 65 mm Hg. | Patients in the LMAP group received a significantly less blood products and total i.v. fluids during intraoperative resuscitation than those in the HMAP group. They had significantly lower mortality in the early postoperative period and a nonsignificant trend for lower mortality at 30 days. | This is a preliminary result publication. The final publication (Matthew et al 2016 (10)) is already included in our meta-analysis. |
| Dewitte 2021 (11) | High mean arterial pressure target to improve sepsis-associated acute kidney injury in patients with prior hypertension: a feasibility study | 26 patients with septic shock | Cross-over of a high-target period (80–85 mmHg) and a low-target period (65–70 mmHg). | In the early stage of sepsis-associated AKI, a high-MAP target in patients with a history of hypertension was associated with a higher CrCl, but did not affect the kidneys' ability to concentrate urine, which may reflect no effect on tubular function | Wrong design (no randomization). |
| Larsson 2018 (12) | Renal effects of norepinephrine-induced variations in mean  arterial pressure after liver transplantation: A randomized  cross-over trial | 10 patients with vasodilation after liver transplantation | Norepinephrine infusion rate was randomly and sequentially titrated tp target MAP levels of 60, 75 and 90 mmHg. | Renal blood flow and GFR are pressure-dependent at MAP levels below 75 mm Hg | Wrong study design. |
| Jakkula 2018 (13) | Targeting low-normal or high-normal  mean arterial pressure after cardiac arrest  and resuscitation: a randomised pilot trial | 120 out of hospital cardiac arrest patients | low‑normal (65–75 mmHg) vs. high‑normal (80–100 mmHg)  MAP | The blood pressure level did not affect the neuron‑specific enolase concentration at 48 h after cardiac arrest, nor any secondary outcomes | No renal outcomes. |
| Hogue 2021 (14) | Personalized Blood Pressure Management  During Cardiac Surgery With Cerebral  Autoregulation Monitoring: A Randomized Trial | 460 cardiopulmonary bypass | MAP targets during  CPB to be above the lower limit of autoregulation or based on usual institutional practice | There was no difference between groups in the frequency of the  composite neurological end-point or its components | Narrow difference of MAP between two arms of intervention. |
| Carrick 2015 (15) | Intraoperative hypotensive resuscitation for patients undergoing laparotomy or thoracotomy for trauma: Early termination of a randomized prospective clinical trial | 168 penetrating trauma patients | Low MAP arm had MAP target at least 50mmHg, high MAP arm was at least 65mmHg | No significant survival advantage existed for the low MAP group at 30 days | No significant different in the average MAP between two arms. |
| Bourgoin 2005 (16) | Increasing mean arterial pressure in patients with septic shock:  Effects on oxygen variables and renal function | 28 septic shock patients | Two groups had MAP maintained at 65mmHg, after that one group had MAP increased to 85mmHg, the other remained 65mmHg for 4h | Increasing MAP from 65 to 85mm Hg with norepinephrine neither affects metabolic variables  nor improves renal function | No relevant outcomes |
| Ameloot 2019 (17) | Early goal-directed haemodynamic  optimization of cerebral oxygenation in  comatose survivors after cardiac arrest:  the Neuroprotect post-cardiac arrest trial | 112 out-of-hospital cardiac arrest patients | early goal directed haemodynamic optimization strategy (MAP 85–100 mmHg, SVO2 65–75%) compared with a MAP 65 mmHg strategy | Targeting a higher MAP in post-CA patients was safe and improved cerebral oxygenation but did not improve the  extent of anoxic brain damage or neurological outcome | No relevant outcomes |
| Lamontagne 2016 (18) | Higher versus lower blood pressure  targets for vasopressor therapy in shock: a  multicentre pilot randomized controlled trial | 118 vasodilatory shock patients | (60–65 mmHg) versus a higher (75–80 mmHg) MAP target | Risks of cardiac arrhythmias (20 versus 36 %, p = 0.07) and hospital mortality (30 versus 33 %, p = 0.84) were not different between lower and higher MAP arms | No relevant outcomes |
| Qiu 2021 (19) | Effects of controlled hypotension with  restrictive transfusion on intraoperative  blood loss and systemic oxygen  metabolism in elderly patients who  underwent lumbar fusion | 40 lumbar fusion surgery | MAP 65mmHg versus MAP 75mmHg | CI, DO2I, and VO2I were lower in both groups at T1–T3 compared with T0 (p < 0.05). DO2I and VO2I in the  MAP 65 group were lower than the MAP 75 group after operation. In both groups, SCysC increased at T1, T2, and T3 (p < 0.05) compared with T0 | No relevant outcomes |
| Damén 2016 (20) | Pressure-dependent changes in haematocrit and plasma volume during anaesthesia, a randomised clinical trial | 24 cardiac surgery patients | MAP at preanaesthesia levels after induction or to a control group that  received vasopressor if MAP decreased below 60 mmHg | haematocrit decreases  and plasma volume increases early and parallel to a decrease in  blood pressure | No relevant outcomes |
| Urzua 1992 (21) | Renal Function and Cardiopulmonary Bypass: Effect of Perfusion Pressure | 21 patients of elective coronary surgery | MAP left untreated in one group, the other MAP > 70mmHg | No deleterious  effect of a low arterial pressure during bypass could be identified. | No relevant outcomes |

# Appendix 8. SUMMARY OF STUDIES AWAITING CLASSIFICATION

| **Author,**  **Identifier** | **Title** | **Participants** | **Intervention** | **Outcomes** | **Main reason** |
| --- | --- | --- | --- | --- | --- |
| Jacques DURANTEAU  ClinicalTrials.gov Identifier: NCT01473498 | Personalized Mean Arterial Pressure Management on Renal Function During Septic Shock (DORESEP) | 27 patients with septic shock | Patients will be treated with fluid and norepinephrine to achieve and maintain a mean arterial pressure of 65 mm Hg. Then they will be randomized in two groups.  -Study group, n=30, mean arterial pressure will be increased to 85 mm Hg for 72 hours by increasing the dose of norepinephrine  -Control group: mean arterial pressure will be maintained at 65 mm Hg. | Acute kidney injury according to RIFLE score. Need for renal replacement therapy. | We could not find the results or progression of this trial registry. |
| Onuigbo MA  2018 | Postoperative AKI: a prospective randomized controlled trial of the impact of two different intraoperative systolic blood pressure levels on renal outcomes | 20‐30 patients in two subsets of patients undergoing noncardiac (orthopaedic) surgery and cardaic (non‐bypass) surgery | Intraoperative target SBP >105 mm Hg vs >85 mm Hg in each arm. | Postoperative AKI | We could not find the results or progression of this trial registry.  <https://www.cochranelibrary.com/es/central/doi/10.1002/central/CN-02215767/full> |
| Bagheri 2012 (22) | The effects of mean arterial pressure during cardiopulmonary bypass on clinical and paraclinical parameters during and after coronary artery bypass graft surgery | 54 patients undergoing CABG surgery with CPB | During CPB, blood pressure was maintained at about 50‐70 mmHg in one group and 70‐90 mmHg in the other group by administration of ephedrine and nitroglycerin | Overall results showed that prognosis in patients with controlled blood pressure at about 70‐90 mmHg during CABG surgery was better compared to the group with a blood pressure of 50‐70 mmHg | This is an conference abstract, we could not find the fulltext. |
| Charlson 2007 (23) | Improvement of Outcomes after Coronary Artery Bypass II: A Randomized Trial Comparing Intraoperative High Versus Customized Mean Arterial Pressure | 412 patients scheduled to undergo primary elective CABG | In one group, mean arterial pressure target during CPB was 80 mmHg (“high” MAP group); in the other group, MAP target was determined by patients’ pre-bypass MAP (“custom” MAP group) | No differences between the high and custom groups in the hours intubated, the hours in the ICU, the days in the hospital, 24-hour blood loss, reoperations for bleeding, acute renal failure or renal dysfunction | Renal data was not numerically available both in full-text and in trial registry. |
| Suk 2006 (24) | Early resuscitation of septic shock to different  level of arterial pressure | 10 patients with septic shock | 65 or 85 mmHg MAP by infusion of norepinephrine | Creatinine clearance  was comparable. | This is an conference abstract. No numerical renal data was available. |

# Appendix 9. SUMMARY OF ONGOING RANDOMIZED CLINICAL TRIALS

| Author,  Identifier | Trial title | Participants | Intervention | Outcomes of interest | Estimated completion date |
| --- | --- | --- | --- | --- | --- |
| Dong-Xin Wang  NCT03629418 | Targeted Blood-pressure Management and Acute Kidney Injury After Coronary Artery Bypass Surgery | 612 Coronary Artery Bypass Surgery | -Experimental:  maintain systolic blood pressure at 110 mmHg or higher during surgery  -Comparator:  maintain systolic blood pressure at 90 mmHg or higher during surgery. | Incidence of acute kidney injury (AKI) within 7 days after surgery | July 2025 |
| Andrew Cochrane  ACTRN12619000128190 | A prospective single centre randomised controlled trial of increased pump flow and arterial pressure during cardiopulmonary bypass to prevent post-operative acute kidney injury. | 400 Patients undergoing cardiac surgery requiring cardiopulmonary bypass | -Usual care  -Experimental: a target mean arterial pressure of at least 80 mmHg | The incidence of stage 1 post-operative acute kidney injury | Sep 2023 |
| Pierre ASFAR  NCT04281277 | Relation Between Mean Arterial Pressure and Renal Resistive Index in the Early Phase of Septic Shock (SEPSIR) | 80 patients with septic shock | -Device: increase of mean arterial pressure at 80-85 mmHg.  -Device: increase of mean arterial pressure at 65-70 mmHg. | changes of KDIGO stage [ Time Frame: Inclusion and day 7 | March 15, 2026 |
| MENGYUN TU   ChiCTR2000037777 | Effect of strict individualized blood pressure management strategy on postoperative acute kidney injury among elderly hypertensive patients undergoing major abdominal surgery | 280  major abdominal surgery | - Experimental group:MAP targeted to remain within ±10% of the reference value using a continuous infusion of norepinephrine.;  -Control group:Continuous infusion of norepinephrine to maintain arterial blood pressure = 80 mmHg.; | Incidence of AKI within the first 7 days after major abdominal surgery.; | 2021-07-30  (not yet recruiting) |
| Yulan Wang  ChiCTR2000041376 | Effect of individualized blood pressure control on troponin and renal function in elderly patients undergoing spinal surgery | 60 spinal surgery patients | -Standard care: Intraoperative MAP is not lower than 60 mmHg or MAP reduction is not more than 40% of the baseline value.  -Intervention: Intraoperative MAP is not lower than 10% of the base value. | Renal function | 2020-12-01 |
| Daniel I Sessler  NCT04884802 | Tight Perioperative Blood Pressure Management to Reduce Serious Cardiovascular, Renal, and Cognitive Complications.  The GUARDIAN Trial | 6254 participants Scheduled for major noncardiac surgery | -Experimental: Tight pressure management  intraoperative MAP ≥85 mmHg, delayed resumption of chronic antihypertensive medications, and a target ward MAP ≥80 mmHg  -Control: Routine pressure management | Composite of major perfusion-related complications | April 25, 2025 |
| Kai Li  NCT04789733 | Tight Perioperative Blood Pressure Management to Reduce Serious Cardiovascular, Renal, and Cognitive Complications: The GUARDIAN Pilot Trial | 80 participants Scheduled for major noncardiac surgery | -Experimental: Tight pressure management  intraoperative MAP ≥85 mmHg, delayed resumption of chronic antihypertensive medications, and a target ward MAP ≥80 mmHg  -Control: Routine pressure management | Perfusion-related complications | June 1, 2024 |
| Bernd Saugel  NCT04894045 | Perioperative Personalized Blood Pressure Management in Patients Having Major Surgery: a Bicentric Prospective Randomized Controlled Interventional Pilot Trial (IMPROVE-pilot) | 200 patients elective major surgery. | -Personalized management group  Intraoperative MAP will be maintained at least at the mean nighttime, and at least at 65 mmHg.  - No Intervention: Control group  Routine intraoperative blood pressure management with a lower intervention threshold of 65 mmHg. | Incidence of acute kidney injury | May 31, 2022 |
| Bingcheng Zhao  NCT04430920 | Blood Pressure Management on Cardiovascular AdveRse Events After Major Abdominal Surgery (BP-CARES): A Randomized Controlled Trial | 1500 participants undergoing major abdominal surgery under general anesthesia | - Intensive intraoperative blood pressure management  Targeting intraoperative mean arterial pressure ≥ 80 mmHg.  - Conventional intraoperative blood pressure management  Targeting intraoperative mean arterial pressure ≥ 65 mmHg or 60% of the baseline level (use the higher target). | Postoperative acute kidney injury | June 2025 |
| Sean van Diepen  NCT04197700 | PRotocolized vs pErsonalized Blood preSSUre peRi-operative paramEters in Coronary Artery Bypass Grafting Surgery: The PRESSURE CABG Cardiac Surgery Trial | 650 participants undergoing non-emergent CABG | -Personalized Arm  Personalized Arm: The target MAP will be defined as +/- 5% of the resting MAP  - Protocolized Arm: The target MAP will be defined as 65 +/- 5mmHg | Composite of delirium or AKI | December 30, 2022 |
| Esteban Poch  NCT04005105 | Acute Post-cardiac Surgery Renal Failure: Prevention Through Individualized Intensive Hemodynamic Management and Evaluation of Prognostic Biomarkers | 240 participants undergoing elective or urgent heart surgery with extracorporeal circulation | - Intensive management  Baseline MAP and central venous pressure will be measured to calculate baseline mean perfusion pressure. Intra-surgical values of ± 25% basal MAP will be maintained  -Standard management  MAP during surgery will be maintained > 60 mmHg according to usual protocol | AKI incidence | December 1, 2022 |
| Chul-Woo Jung | Comparison of Individualized vs Standard Blood Pressure Target on the Postoperative Myocardial Injury in High Risk Patients Undergoing Non-cardiac Surgery- a Randomized Non-inferiority Trial | 420 participants scheduled for general or urologic surgeries | - Standardized blood pressure management with a target of mean blood pressure greater than 65mmHg and systolic blood. pressure lower than 160mmHg  -Individualized  Individualized blood pressure management of 20% within the preoperative ward blood pressure. | acute kidney injury | May 31, 2022 |
| Jesper Kjaergaard  NCT03141099 | Blood Pressure and Oxygenation Targets in Post-resuscitation Care, a Randomized Clinical Trial | 800  comatose out-of-hospital cardiac arrets patients | -Low normal MAP and low normal PaO2  MAP 63 mmHg and PaO2 9-10 kPa during targeted temperature management (36 hours) after OHCA  - High normal MAP and low normal PaO2  MAP 77 mmHg and PaO2 9-10 kPa  Low normal MAP and high normal PaO2  MAP 63 mmHg and PaO2 13-14 kPa  High normal MAP and high normal PaO2  MAP 77 mmHg and PaO2 13-14 kPa | Renal replacement therapy | December 2021 |
| Yasuhiro Otomo  UMIN000041775 | Optimal Target Blood Pressure in Elderly with Septic Shock trial (OPTPRESS trial) | 836 patients with septic shock | -Targeting mean arterial pressure of 80-85 mmHg during the first 72 hours after randomization  -Targeting mean arterial pressure of 65-70 mmHg during the first 72 hours after randomization | Renal replacement therapy-free days at 28 days after randomization | 31/12/2024 |
| Maiwall 2021 (25) | High versus low target mean arterial pressure in managing septic shock in critically ill cirrhosis patients - a prospective open-label randomized controlled trial | 150 septic shock patients with cirrhosis | high MAP (80-85 mm of Hg) versus low MAP (60-65 mm | Rate of AKI, RRT receipt rate | This studied has been completed but not yet to be published |

# Appendix 10. SUMMARY OF OUTCOMES REPORTED IN THE INCLUDED STUDIES AND DEFINITIONS USED FOR AKI

| **Study ID** | **Primary outcome** | **Secondary outcome** | **Renal outcomes used in this review** | **Renal outcomes not used in this review** | **AKI definitions and data conversion** |
| --- | --- | --- | --- | --- | --- |
| **Non-surgery studies** | | | | | |
| Asfar  2014 | 1.Death from any cause by 28 days | 1. 90-day mortality  2. Days alive and free from organ dysfunction by day 28  3. Length of stay in the intensive care unit and hospital. | 1. Doubling of plasma creatinine during days on vasopressor  2. RRT receipts from day 1 to day 7  3. Cumulative urine output from day 1 to day 5 (liters) | 1. Daily urine output day 1-5 | Doubling of plasma creatinine during days on vasopressor rates were equivalent to RIFLE stage 2 criteria. |
| Grand  2020 | 1. Plasma concentration of soluble thrombomodulin (sTM) after 48 h | 1. Biomarkers indicative of endothelial activation, circulating biomarkers of cell death  2. The haemostatic profile 3. Levels of neuron-specific enolase 48h post-inclusion. | 1. Day 3 urine output (ml)  2. Day 3 eGFR (ml/min/1.73m2)  3. RRT rates within 4 days. | 1. Day 1, 2 urine output (ml)  2. Day 1, 2 eGFR (ml/min/1.73m2) |  |
| Lamontagne 2020 | 1. 90-day mortality | 1. Mortality at discharge from the critical care unit and acute hospital,  2. Duration of survival to longest available follow-up.  3. Duration of advanced respiratory and renal support  4. Duration of critical care unit and acute hospital stay  5. Cognitive decline at 90 days and 1 year. | 1. Severe acute renal failure (KDIGO stage 3 criteria) from randomization until critical care discharge  2. RRT rates within 28 days.  3. mean daily urine output (ml/kg/hour) during the  first episode of vasopressor treatment | 1. days on RRT, days alive and free of renal support |  |
| **Non-cardiac surgery studies** | | | | | |
| Futier 2017 | 1. Composite of systemic inflammatory response syndrome and at least 1 organ system dysfunction by day 7 after surgery | 1. The individual components of the primary composite outcome;  2. Changes in hemodynamic variables; the SOFA score on days 1, 2, and 7; the SIRS score; postoperative complications; durations of intensive care unit and hospital stay; and all-cause mortality at 30 days after surgery. | 1. AKI according to RIFLE “risk” stage within 7 days  2. RRT rates within 30 days | 1. AKI according to RIFLE “Injury” and “Failure”  1. RRT rates within 7 days |  |
| Hu 2021 | 1. Incidence of post-operative delirium | 1. length of PD during the 7-day followup period, intraoperative urine volume, intraoperative blood loss, and  occurrence of non-delirium postoperative complications (bleeding  requiring intervention, new arrhythmias, heart failure, liver failure,  kidney injury, respiratory failure, or infection). | 1. AKI defined by the KDIGO creatinine-based criteria within 7 days.  2. Intraoperative urine volume ml/kg/h | - |  |
| Wanner 2021 | 1. Composite of hs-cTnI rise on POD 0-3 and/or 30-day MACE/acute kidney injury (AKI) | 1. 1-year MACE, a composite identical to 30-day MACE/AKI | 1. AKI AKIN criteria as an abrupt (within 48 hours) reduction in kidney function within 30 days  2. CKD at 1 year | - |  |
| Wu 2017 | 1. Incidence of AKI after major abdominal surgery during the first 7 postoperative days. | 1. Incidence of surgical site infection, hospital-acquired pneumonia, stroke, admission to the ICU, stay in the ICU, length of hospital stay, and 28-day mortality. | 1. AKI according to KDIGO any stages within 7 days postoperative. | 1. AKI KDIGO stage 1, 2, 3 within 7 days postoperative. |  |
| **Cardiac surgery studies** | | | | | |
| Azau 2014 | 1. Rates of AKI | 1. Renal function at day 28 and at six months after surgery, death rate. | 1. AKI classified as RIFLE “risk”  2. RRT receipts  3. Serum creatinine peak  4. Mean hourly diuresis (ml/kg/h)  All within 5 days postoperative. | 1. AKI according to 30% rise in serum creatinine  2. AKI according to 50% rise in serum creatinine  3. AKI classified as RIFLE “Injury”  4. Numer of dialysis/patient in ICU when required  5. Time of occurrence of serum creatinine peak  6. Renal resistivity index |  |
| Kandler 2019 | mean change in glomerular filtration rate (GFR) at follow-up compared to baseline | change in urinary Neutrophil Gelatinase-Associated Lipocalin (uNGAL) and | 1. AKI RIFLE criteria. In accordance with the RIFLE criteria an eGFR decrease of > 25% was also used to define AKI.  2. Dialysis postoperatively (time frame not clear)  3. Urine output intraoperative  4. Delta serum creatinine levels (mmol/L) within 48 hours.  5. Delta eGFR (ml/min) within 48 hours.  6. Change in GFR (ml/min) 4 months follow-up  7. >10% decrease in GFR 4 months follow up. | 1.uNGAL/creatinine changes from baseline (ng/mL) within 120 hours. |  |
| Siepe 2011 | 1. Cognitive outcome as measured by the Mini-Mental-State examination (MMSE) before and 48 h after surgery | 1. Complications, use of blood products, urinary output, maximum weight gain, ventilation time, and ICU- and total postoperative stay were documented according to the patients’ records | 1. AKI (definition not provided) within 7 days postoperative.  2. Urine output first 24 hour postoperative (ml)  3. Creatinin levels day 7. | 1. Creatinin levels day 1, 2 and highest measured value postoperative. |  |
| Sirvinskas 2012 | 1. Renal function in elderly patients  during the early postoperative period |  | 1. Acute renal failure (ARF) in the postoperative period was defined according to ADQI Group with reference to RIFLE. Reported as oliguria rate. | 1. Urine excretion |  |
| Vedel 2018 | 1. Total volume of new ischemic lesions, expressed as the difference between DWI conducted preoperatively and again between days 3 and 6 | 1. Total number of new ischemic cerebral lesions  Postoperative cognitive dysfunction and new focal neurological deficits, both evaluated as a change from baseline neuropsychological and neurological test performance to the result at 1 week, at discharge from the hospital, or at healthcare relocation from the cardiac surgery ward to a local hospital | 1. Rates of doubling of creatinine level baseline value during hospital stay  2. Postoperative dialysis within 30 days  3. Creatinine levels peak value first 24 hours during hospital stay | - | Rates of doubling of creatinine level baseline value during hospital stay rates were equivalent to RIFLE stage 2 criteria |

# Appendix 11a. SUMMARY OF CHARACTERISTICS OF THE INCLUDED STUDIES

| **Group of**  **study** | **Study ID** | **Study design** | **Follow-up duration** | **Setting of hypotension** | **Number of participants**  **(Intention to treat)** | **Inclusion criteria**  **(fitting all the criteria)** | **Major exclusion criteria**  **(fitting any of the criteria)** | **Recruiting sites and Funding** |
| --- | --- | --- | --- | --- | --- | --- | --- | --- |
| Shock | Asfar  2014 (7) | Multicenter RCT | 90 days | Septic shock | 776 | 1. Patients older than 18 years of age  2. Had septic shock that was refractory to fluid resuscitation  3. Required vasopressors infusion  4. Were evaluated within 6 hours after the initiation of vasopressors. | 1. Legal protection  2. No affiliation with the French health care system, pregnancy  3. Recent participation in another biomedical study or another interventional  4. Investigator decided not to resuscitate. | - 29 centers in France.  - Funded by the French Ministry of Health. |
|  | Grand  2020 (26) | Single-center, double-blinding RCT | 180 days | OHCA patients | 50 | Adult (≥18 years), comatose (Glasgow Coma Score ≤8)  resuscitated OHCA patients of presumed cardiac cause  with sustained return of spontaneous circulation for >20 min, irrespective of the initial rhythm. | 1. Unwitnessed OHCA with asystole as primary rhythm.  2. Suspected or confirmed acute intracranial bleeding,  3. >240 min from return of spontaneous circulation to randomization.  4. SBP <80mm Hg despite of fluid administration.  5. Vasopressors, inotropic support and/or mechanical assist device.  6. Temperature on admission <30°C.  7. Pregnancy. | - Department of Cardiology, Rigs hospital Denmark  - Funded by: none. |
|  | Lamontagne 2020 (27) | Multicenter RCT | 1 year | Vasodilatory shock | 2463 | 1. Patients were aged ≥ 65 years.  2. Patients had vasodilatory hypotension  3. Patients had started an infusion (for at least 1 hour) of vasopressors within the prior 6 hours  4. Patients had adequate fluid resuscitation completed or ongoing.  5. Vasopressors were expected to be continued for ≥ 6 further hours. | 1. Vasopressors being used solely as therapy for bleeding  2. Acute ventricular failure (left or right)  3. Post-cardiopulmonary bypass vasoplegia.  4. Ongoing treatment for brain injury or spinal cord injury.  5. Death perceived as imminent.  6. Previous enrolment to the 65 trial. | -65 sites adult general ICUs across England, Wales and Northern Ireland.  - Funded by: the National Institute for Health Research Health Technology Assessment program |
| Non-cardiac surgery | Futier  2017 (28) | Multicenter RCT | 30 days after surgery | Major surgery | 292 | 1. 50 years or older  2. Scheduled to undergo surgery under general anesthesia with an expected duration of 2 hours or longer.  3. Had an American Society of Anesthesiologists physical status of class II or higher.  4. Had a preoperative  acute kidney injury risk index14 of class III or higher. | 1. Had severe uncontrolled hypertension.  2. Had chronic kidney disease.  3. Had acute or decompensated heart failure or acute coronary syndrome.  4. Had preoperative sepsis or were already receiving norepinephrine infusion.  5. Required renal vascular surgery. | -9 French university and nonuniversity hospitals.  - Funded by: the  University Hospital of Clermont-Ferrand and in part by a grant from Aguettant. |
|  | Hu  2021 (29) | Multicenter RCT | 7 days after surgery | Non-cardiothoracic surgery | 298 | 1. Patients ≥65 years of age with an American Society of Anesthesiologists (ASA) physical status of I-II  2. Underwent non-cardiothoracic surgery with GA with an anticipated length of surgery time of at least 2h | 1. History of schizophrenia,  epilepsy, parkinsonism, diabetes, hypertension, severe sinus bradycardia  2. Second-degree or greater atrioventricular block without a pacemaker; use of a cholinesterase inhibitor  or levodopa  3. Severe hepatic dysfunction, severe renal dysfunction (dialysis before surgery)  4. Brain injury or previous  Neurosurgery.  5. Severe cognitive impairment. | - 7 hospitals in China.  - Funded by the National Science Foundation for  Young Scientists of China  and the Shenzhen Key Medical Discipline Construction  Fund. |
|  | Wanner  2021 (30) | Single-center RCT | 1 year after surgery | Major noncardiac  surgery | 451 | Patients over 45 years of age at cardiovascular risk undergoing major noncardiac surgery | 1. Pregnancy.  2. Inclusion in  another clinical trial with common endpoints  3. Emergent surgery,  presence of any active cardiac conditions.  4. Any transplantation. | - Cantonal  Hospital St. Gallen, Switzerland.  - Funded from the Swiss National ScienceFoundation, the Swiss Heart Foundation and the Scientific Commission. |
|  | Wu  2017 (31) | Multicenter RCT | 28 days | Elective major gastrointestinal surgery | 646 | 1. Patients with chronic hypertension.  2. Scheduled for elective major gastrointestinal surgery.  3. 65–80 years of age;  4. Patients had American Society Anesthesiologists physical status grade I to III disease; | 1. Patients used non-steroidal anti-inflammatory drugs during the past month;  2. Patients had heart failure during the past 2 months;  3. Patients had myocardial infarction during the past month (confirmed by blood-specific enzymes); 4. Current severe pulmonary function insufficiency;  5. Current intermediate to severe pulmonary hypertension;  6. Chronic kidney diseases or renal dysfunction | - 3 teaching hospitals in China  - Source of funding: Only department support. |
| Cardiac surgery | Azau  2014 (32) | Single-center RCT | 6 months | elective cardiac surgery | 292 | 1. Elective cardiac surgery under normothermic CPB.  2. Patients with  known risk factors for acute kidney injury. | 1. Infusion of a radiocontrast agent or treatment by a nephrotoxic agent one and three weeks before surgery respectively.  2. Chemotherapy within the three last months.  3. Liver cirrhosis, heart failure, renal artery stenosis, pulmonary hypertension.  4. Patients who eventually  disclosed a major perioperative complication (shock, emergent re-operation), identified as AKI cause. | -LUNAM university hospital, France.  -Source of funding: the “Programme  Hospitalier pour la Recherche Clinique” from the French Health Ministry |
|  | Kandler 2019 (33) | Single-center RCT | 6 months | Cardiopulmonary bypass | 90 | Patients > 70 years old and complex cardiac surgery procedures | 1. Serum creatinine (sCr) > 200 μmol/L  2. Previous heart surgery, endocarditis and acute operation defined as coronary angiography < 24h of surgery. | -Rigshospitalet Copenhagen University Hospital,  Denmark  -Funded by The Danish Heart Foundation |
|  | Siepe  2011 (34) | Single-center RCT | 7 days after surgery. | CABG | 92 | On-pump elective or urgent CABG | 1. Patients with a history of cerebrovascular diseases or previous psychiatric  disorders  2. Emergency surgery, preoperative instability, concomitant surgery.  3. Patients incapable of taking the neuropsychological test.  4. Desired pressure during CPB was not achieved or postoperative cognitive evaluation was impossible. | -University Medical Center Freiburg, Germany  -Source of funding: Not mentioned. |
|  | Sirvinskas 2012 | Single-center RCT | 3 days after surgery. | CABG surgery on CPB | 122 | 1. elderly patients (70 years or older)  2. Normal preoperative renal function | 1. Patients with diabetes mellitus | Department of Cardiothoracic and Vascular Surgery of Hospital of  Lithuanian University of Health Sciences, Kaunas, Lithuania |
|  | Vedel  2018 (35) | Single-center RCT | 2-4months | elective or subacute onpump coronary artery bypass grafting and/or left-sided heart  valve surgery | 197 | 1. Patients ≥18 years of age  2. Were in need of elective or subacute onpump coronary artery bypass grafting and/or left-sided heart  valve surgery | 1. Pre-existing continuous renal replacement therapy or those initiated on dialysis during their hospital stay prior to enrollment in  the study.  2. Artificial liver support therapies  3. Ongoing gastrointestinal bleeding  4. Active medical disease requiring surgical intervention  5. Pre-existing or in-hospital placement of transjugular intrahepatic portosystemic shunt  6. Long standing hypertension (on active treatment)  7. Improvement in renal function after central blood volume expansion  8. Contraindications to norepinephrine (active myocardial event, ventricular arrhythmia, obstructive physiology, limb ischemia) | - A university hospital in the capital region of Denmark.  - Source of funding: the Danish Heart Foundation and the Research Foundations at  Rigshospitalet, University of Copenhagen, Denmark. |

# Appendix 11b. SUMMARY OF CHARACTERISTICS OF THE INCLUDED STUDIES (CONTINUED)

| **Group of study** | **Study ID** | **Lower BP target arm** | | **Higher BP target arm** | | **Methods protocolized to reach BP target** |
| --- | --- | --- | --- | --- | --- | --- |
|  |  | **Target** | **Average time of intervention** | **Target** | **Average time of intervention** |  |
| Shock | Asfar 2014 (7) | MAP 65-70mmHg | 5 days | MAP 80-85mmHg | 5 days | Fluid resuscitation, norepinephrine. Use of activated protein C and hydrocortisone was left to the discretion of the attending physician. |
|  | Grand 2020 (26) | MAP 65mmHg | 48 hours | MAP 72mmHg | 48 hours | No predefined method on how the BP target should be achieved. |
|  | Lamontagne 2020 (27) | MAP 60-65mmHg | Median (IQR):  33.0 (15.0 to 56.0) hours | Usual care  MAP median (IQR):  72.6 (69.4 to 76.5) | Median (IQR):  38.0 (19.0 to 67.0) hours | Vasopressors titration. Choice of vasopressor as well as all other interventions were also at the discretion of treating clinicians. |
| Non-cardiac surgery | Futier 2017 (28) | SBP not lower than 80mmHg or 40% of patient’s reference value.  (MAP=75±13mmHg) | Median (IQR) 465 (390-600) minutes | SBP remained within ±10% of the reference value  (MAP=81±14mmHg) | Median (IQR) 423 (342-550) minutes | Protocol of fluid infusion and norepinephrine titration. |
|  | Hu 2021 (29) | MAP 60-70mmHg | Median (IQR) 228 (189–252) min | MAP 95-100mmHg | Median (IQR) 211 (188–251) min | Titration of intravenous antihypertensives and phenylephrine. Fluid and blood products infusion. |
|  | Wanner 2021 (30) | MAP ≥60 mm Hg | Median (IQR) 5.4 (4,3-7,0) hours* | MAP ≥75 mm Hg | Median (IQR) 5.3 (4,2-7,1) hours* | Clinical implementation of the institutional hemodynamic management algorithm was at the discretion of the anesthesiologist in charge. |
|  | Wu 2017 (31) | Level I MAP 65-79mmHg | 220.6 ± 71.0 mins | Level II MAP 80-95mmHg and level III MAP 96-110mmHg | Level II 212.9 ± 73.6 and level III 218.9 ± 69.2 mins | Vasoactive agents and nitroglycerin. |
| Cardiac surgery | Azau 2014 (32) | MAP 50-60mmHg | 113 ± 51 mins | MAP 75-85mmHg | 118 ± 43 mins | Norepinephrine infusion. |
|  | Kandler 2019 (33) | Standard care  (47 ± 5mmHg) | 130 ± 36 mins | High arterial pressure  (>60mmHg) | 130 ± 31 mins | Infusion of fluid and norepinephrine. |
|  | Siepe 2011 (34) | MAP 60-70mmHg | 101 +25 mins | MAP 80-90mmHg | 91 +30 mins | Norepinephrine infusion and boli of urapidil. |
|  | Sirvinska 2012 | MAP <60 mmHG | 90.1±28.9 | MAP 60-70mmHg  MAP >70mmHG | 109.5±43.7  109.7±45.3 | MABP was controlled using catecholamines (norepinephrine)  or nitrates |
|  | Vedel 2018 (35) | MAP 40-50mmHg | 94.0±33.0 mins | MAP 70-80mmHg | 105.6±77.4 mins | Intermittent intravenous doses of phenylephrine, followed by infusion of norepinephrine. |

# Appendix 12. AVAILABLE BASELINE CHARACTERISTICS OF PATIENTS IN THE INCLUDED STUDIES

| **Group of study** | **Study ID** | **Age (years)**  **mean±SD or median (IQR)** | | **Male sex (n(%))** | | **Premorbid hypertension** | | | **Baseline renal functions** | | |
| --- | --- | --- | --- | --- | --- | --- | --- | --- | --- | --- | --- |
|  |  | **Low BP** | **High BP** | **Low BP** | **High BP** | **Low BP** | **High BP** | **Reported features** | | **Low BP** | **High BP** |
| Non-surgery | Asfar 2014 | 65±15 | 65±13 | 64.4% | 68.8% | 44.6% | 43.0% | -Chronic kidney disease  -Chronic kidney disease requiring long-term dialysis  -Acute kidney injury  -Serum creatinine (mg/dl) | | 7.7%  3.1%  48.7%  1.96±1.39 | 5.2%  1.3%  45.1%  1.93±1.47 |
|  | Grand 2020 | 59 ±13 | 63 ±10 | 92% | 83% | 38% | 43% | -Nephropathy | | 23% | 22% |
|  | Lamontagne 2020 | 75.2 (70.4-80.5) | 74.8 (70.1-80.8) | 57.2% | 55.8% | 46% | 46% | -Chronic renal replacement therapy at ICU admission | | 1.3% | 1.5% |
| Non-cardiac surgery | Futier 2017 | 70.0±7.5 | 69.7±7.1 | 84.8% | 85% | 82.8% | 81.8% | -Renal impairment  -Serum creatinine (mg/dl) | | 11.7%  0.93±0.34 | 19.1%  0.93±0.30 |
|  | Hu 2021 | 73 (68–77) | 72 (68–77) | 39.9% | 43.2% | 0% | 0% | - | | - | - |
|  | Wanner 2021 | 69 ± 10 | 70±8 | 81% | 83% | - | - | -Preoperative serum creatinine >175 mmol/L | | 15% | 15% |
|  | Wu 2017 | 73 ± 7 | Level 2  73 ± 6  Level 3  74 ± 5 | 60.2% | Level 2  68.3%  Level 3  65.6% | 100%** | 100%** | -Serum creatinine (mmol/L) | | 54.3 ± 7.9 | Level 2  52.9 ± 5.7  Level 3  53.8 ± 6.7 |
| Cardiac surgery | Azau 2014 | 76 ± 7 | 76 ± 8 | 73.1% | 63.9% | - | - | -Serum creatinine clearance between 30 and 60 ml/min/1.73m² | | 50% | 57% |
|  | Kandler 2019 | 76.3 ± 4.2 | 76.6 ± 4.7 | 73% | 71% | 80% | 68% | -GFR (ml/min)  -sCr (μmol/dl) median (IQR)  -eGFR (ml/min) | | 77 ± 21  88 (80–101)  72 ± 22 | 69 ± 28  84 (69–116)  71 ± 29 |
|  | Siepe 2011 | 65.2 ±9.6 | 68.7±8.3 | 83% | 77% | 63% | 72% | -Severe renal insufficiency | | 10% | 16% |
|  | Sirvinska 2012 | 74.3±3.6 | 75.3±4.2  74.9±4.1 | 52.8% | 58%  44.4% | 69.4% | 70%  80.6% | - | |  |  |
|  | Vedel 2018 | 65.0±10.7 | 69.4±8.9 | 93.9% | 85.7% | 84.8% | 88.8% | -Plasma creatinine mg/dl | | 1.05±0.32 | 1.01±0.20 |

# Appendix 13. RISK OF BIAS ASSESSMENT OF THE INCLUDED STUDIES


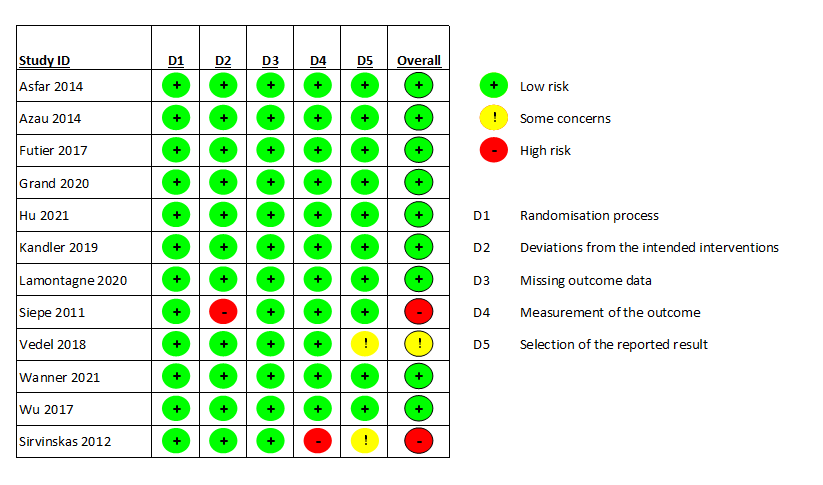


# Appendix 14. SUMMARY OF FINDINGS AND LEVEL OF CERTAINTY ASSESSMENT OF THE BODY OF EVIDENCE

A. Higher MAP compared to normotension for shock patients to prevent AKI progression

| **Certainty assessment** | | | | | | | **№ of patients** | | **Effect** | | **Certainty** | **Importance** |
| --- | --- | --- | --- | --- | --- | --- | --- | --- | --- | --- | --- | --- |
| **№ of studies** | **Study design** | **Risk of bias** | **Inconsistency** | **Indirectness** | **Imprecision** | **Other considerations** | **Higher MAP** | **normotension** | **Relative (95% CI)** | **Absolute (95% CI)** |  |  |
| **AKI rate** | | | | | | | | | | | | |
| 2 | randomised trials | not serious | not serious | not serious | not serious^a^ | none | 183/1688 (10.8%) | 202/1671 (12.1%) | **RR 1.10** (0.93 to 1.29) | **12 more per 1.000** (from 8 fewer to 35 more) | ⨁⨁⨁⨁ High | CRITICAL |
| **RRT receipt rate** | | | | | | | | | | | | |
| 3 | randomised trials | not serious | not serious | not serious | not serious^a^ | none | 439/1650 (26.6%) | 449/1632 (27.5%) | **RR 1.03** (0.92 to 1.16) | **8 more per 1.000** (from 22 fewer to 44 more) | ⨁⨁⨁⨁ High | CRITICAL |
| **RRT receipt rate in hypertension patient** | | | | | | | | | | | | |
| 2 | randomised trials | not serious | not serious | not serious | serious^b^ | none | 193/735 (26.3%) | 231/731 (31.6%) | **RR 1.20** (1.03 to 1.41) | **63 more per 1.000** (from 9 more to 130 more) | ⨁⨁⨁◯ Moderate | CRITICAL |
| **RRT receipt rate in non-hypertension patient** | | | | | | | | | | | | |
| 2 | randomised trials | not serious | not serious | not serious | serious^b^ | none | 243/892 (27.2%) | 210/875 (24.0%) | **RR 0.88** (0.75 to 1.03) | **29 fewer per 1.000** (from 60 fewer to 7 more) | ⨁⨁⨁◯ Moderate | CRITICAL |

**CI: confidence interval; RR: risk ratio**

**Explanations**

**a. Not reaching Optimal Information Size but large sample (>2000 patients)**

**b. Not reaching Optimal Information Size**

B. Higher MAP compared to normotension for cardiac surgery patients to prevent AKI

| **Certainty assessment** | | | | | | | **№ of patients** | | **Effect** | | **Certainty** | **Importance** |
| --- | --- | --- | --- | --- | --- | --- | --- | --- | --- | --- | --- | --- |
| **№ of studies** | **Study design** | **Risk of bias** | **Inconsistency** | **Indirectness** | **Imprecision** | **Other considerations** | **Higher MAP** | **normotension** | **Relative (95% CI)** | **Absolute (95% CI)** |  |  |
| **AKI rate** | | | | | | | | | | | | |
| 4 | randomised trials | serious^a^ | serious^b^ | not serious | serious^c^ | none | 132/418 (31.6%) | 104/372 (28.0%) | **RR 0.87** (0.73 to 1.03) | **36 fewer per 1.000** (from 75 fewer to 8 more) | ⨁◯◯◯ Very low | CRITICAL |
| **RRT receipt rate** | | | | | | | | | | | | |
| 3 | randomised trials | serious^a^ | not serious | not serious | very serious^c,d^ | none | 11/374 (2.9%) | 10/324 (3.1%) | **RR 0.92** (0.39 to 2.14) | **2 fewer per 1.000** (from 19 fewer to 35 more) | ⨁◯◯◯ Very low | CRITICAL |

**CI:** confidence interval; **RR:** risk ratio

#### Explanations

a. Most studies have high or some-concern RoB

b. Inconsistent effect in random-effect meta-analysis

c. Not reaching Optimal Information Size

d. Very wide confident interval

C. Higher MAP compared to normotension for non-cardiac surgery patients to prevent AKI

| **Certainty assessment** | | | | | | | **№ of patients** | | **Effect** | | **Certainty** | **Importance** |
| --- | --- | --- | --- | --- | --- | --- | --- | --- | --- | --- | --- | --- |
| **№ of studies** | **Study design** | **Risk of bias** | **Inconsistency** | **Indirectness** | **Imprecision** | **Other considerations** | **Higher MAP** | **normotension** | **Relative (95% CI)** | **Absolute (95% CI)** |  |  |
| **AKI rate** | | | | | | | | | | | | |
| 4 | randomised trials | not serious | not serious | not serious | serious^a^ | none | 159/943 (16.9%) | 173/744 (23.3%) | **RR 1.25** (0.98 to 1.60) | **58 more per 1.000** (from 5 fewer to 140 more) | ⨁⨁⨁◯ Moderate | CRITICAL |
| **RRT rate** | | | | | | | | | | | | |
| 1 | randomised trials | not serious | not serious | not serious | very serious^a,b^ | none | 6/147 (4.1%) | 7/145 (4.8%) | **RR 1.18** (0.41 to 3.43) | **9 more per 1.000** (from 28 fewer to 117 more) | ⨁⨁◯◯ Low | CRITICAL |

**CI:** confidence interval; **RR:** risk ratio

#### Explanations

a. Not reaching Optimal Information Size

b. Very wide confident interval

# Appendix 15. S-Figure 1. FUNNEL PLOT OF INCLUDED STUDY WITH OUTCOME OF AKI RATE

**
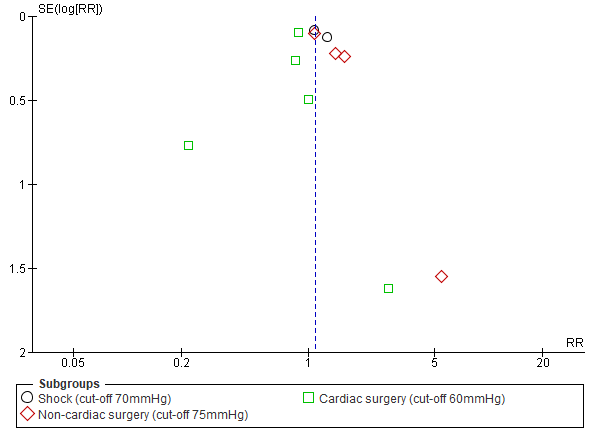
**

# Appendix 16. S-Figure 2. Higher MAP versus normotension in shock patients without hypertension

**
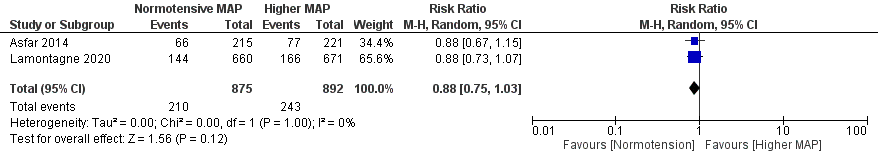
**

# Appendix 17. S-Figure 3. Meta-regression of Log risk ratio of RRT rate on Age


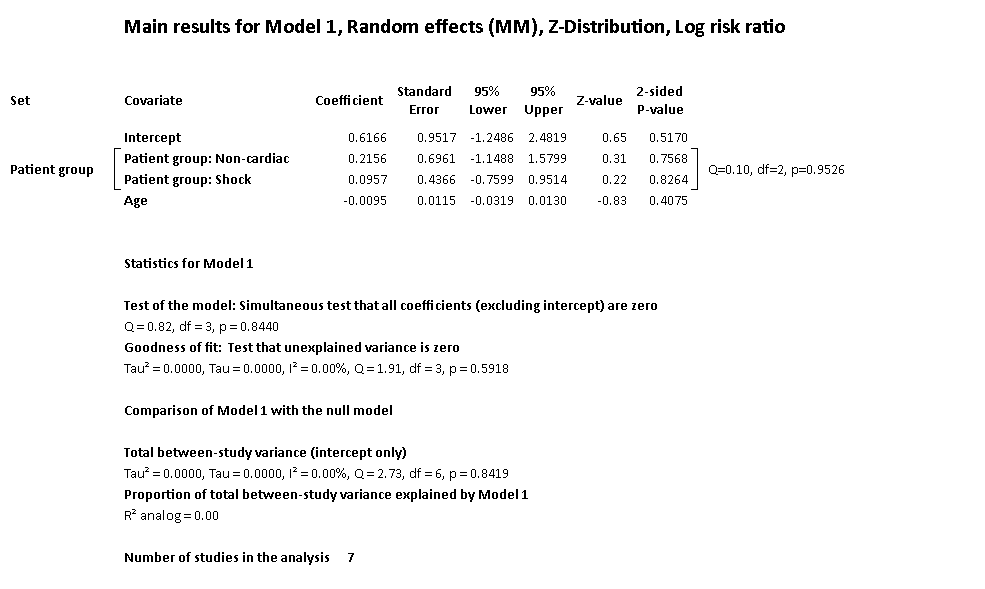

# Appendix 18. S-Figure 4. Meta-regression of Log risk ratio of RRT rate on Hypertension percentage and RoB
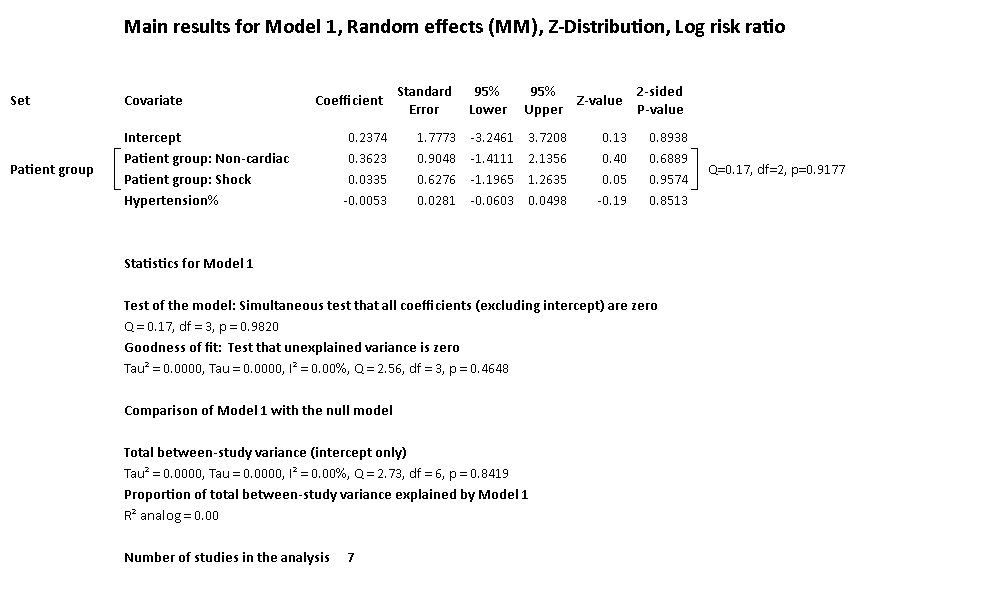

Appendix 19. S-Figure 5. Meta-regression of Log risk ratio of RRT rate on RoB


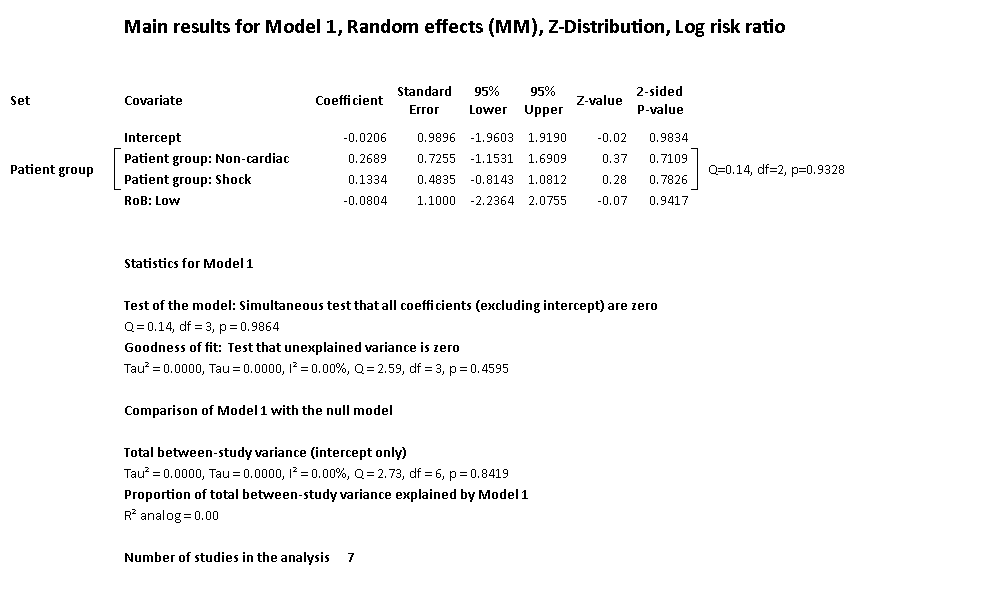

# REFERENCES

1. Varajic B, Cavallazzi R, Mann J, Furmanek S, Guardiola J, Saad M. High versus low mean arterial pressures in hepatorenal syndrome: A randomized controlled pilot trial. *J Crit Care*. 2019;52:186-92.

2. Karavana V, Smith I, Kanellis G, Sigala I, Kinsella T, Zakynthinos S, et al., editors. 37th International Symposium on Intensive Care and Emergency Medicine (part 1 of 3). Critical Care; 2017: BioMed Central.

3. Redfors B, Bragadottir G, Sellgren J, Swärd K, Ricksten S-EJIcm. Effects of norepinephrine on renal perfusion, filtration and oxygenation in vasodilatory shock and acute kidney injury. 2011;37(1):60-7.

4. Shao Z, Du Z, Wang R, Wang Z, He X, Wang H, et al. Effects of different target blood pressure resuscitation on peripheral blood inflammatory factors and hemodynamics in patients with traumatic hemorrhagic shock. 2019;31(4):428-33.

5. Gold JP, Charlson ME, Williams-Russo P, Szatrowski TP, Peterson JC, Pirraglia PA, et al. Improvement of outcomes after coronary artery bypass. A randomized trial comparing intraoperative high versus low mean arterial pressure. *J Thorac Cardiovasc Surg*. 1995;110(5):1302-11; discussion 11-4.

6. Rose A, Owens R, Malhotra A, Asfar P. The Impact of High Versus Low Mean Arterial Pressure Goals in Cirrhotic Patients with Septic Shock. D104. CRITICAL CARE: A FINE BALANCE-SEPSIS DEFINITIONS, OUTCOMES AND EPIDEMIOLOGY: American Thoracic Society; 2019. p. A7155-A.

7. Asfar P, Meziani F, Hamel J-F, Grelon F, Megarbane B, Anguel N, et al. High versus Low Blood-Pressure Target in Patients with Septic Shock. *N Engl J Med*. 2014;370(17):1583-93.

8. Bootsma IT, de Lange F, Scheeren TW, Jainandunsing JS, Boerma ECJJoc, anesthesia v. High Versus Normal Blood Pressure Targets in Relation to Right Ventricular Dysfunction After Cardiac Surgery: A Randomized Controlled Trial. 2021.

9. Morrison CA, Carrick MM, Norman MA, Scott BG, Welsh FJ, Tsai P, et al. Hypotensive resuscitation strategy reduces transfusion requirements and severe postoperative coagulopathy in trauma patients with hemorrhagic shock: preliminary results of a randomized controlled trial. 2011;70(3):652-63.

10. Carrick MM, Morrison CA, Tapia NM, Leonard J, Suliburk JW, Norman MA, et al. Intraoperative hypotensive resuscitation for patients undergoing laparotomy or thoracotomy for trauma: Early termination of a randomized prospective clinical trial. *J Trauma Acute Care Surg*. 2016;80(6):886-96.

11. Dewitte A, Labat A, Duvignaud PA, Bouche G, Joannes-Boyau O, Ripoche J, et al. High mean arterial pressure target to improve sepsis-associated acute kidney injury in patients with prior hypertension: a feasibility study. *Ann Intensive Care*. 2021;11(1):139.

12. Skytte Larsson J, Bragadottir G, Redfors B, Ricksten SE. Renal effects of norepinephrine-induced variations in mean arterial pressure after liver transplantation: A randomized cross-over trial. *Acta Anaesthesiol Scand*. 2018;62(9):1229-36.

13. Jakkula P, Pettilä V, Skrifvars MB, Hästbacka J, Loisa P, Tiainen M, et al. Targeting low-normal or high-normal mean arterial pressure after cardiac arrest and resuscitation: a randomised pilot trial. *Intensive Care Med*. 2018;44(12):2091-101.

14. Hogue CW, Brown CHt, Hori D, Ono M, Nomura Y, Balmert LC, et al. Personalized Blood Pressure Management During Cardiac Surgery With Cerebral Autoregulation Monitoring: A Randomized Trial. *Semin Thorac Cardiovasc Surg*. 2021;33(2):429-38.

15. Carrick MM, Morrison CA, Tapia NM, Leonard J, Suliburk JW, Norman MA, et al. Intraoperative hypotensive resuscitation for patients undergoing laparotomy or thoracotomy for trauma: Early termination of a randomized prospective clinical trial. *Journal of Trauma and Acute Care Surgery*. 2016;80(6):886-96.

16. Bourgoin A, Leone M, Delmas A, Garnier F, Albanèse J, Martin C. Increasing mean arterial pressure in patients with septic shock: Effects on oxygen variables and renal function*. *Crit Care Med*. 2005;33(4):780-6.

17. Ameloot K, De Deyne C, Eertmans W, Ferdinande B, Dupont M, Palmers PJ, et al. Early goal-directed haemodynamic optimization of cerebral oxygenation in comatose survivors after cardiac arrest: the Neuroprotect post-cardiac arrest trial. *Eur Heart J*. 2019;40(22):1804-14.

18. Lamontagne F, Meade MO, Hébert PC, Asfar P, Lauzier F, Seely AJE, et al. Higher versus lower blood pressure targets for vasopressor therapy in shock: a multicentre pilot randomized controlled trial. *Intensive Care Med*. 2016;42(4):542-50.

19. Qiu X, Tan Z, Tang W, Ye H, Lu X. Effects of controlled hypotension with restrictive transfusion on intraoperative blood loss and systemic oxygen metabolism in elderly patients who underwent lumbar fusion. *Trials*. 2021;22(1):99.

20. Damén T, Reinsfelt B, Redfors B, Nygren A. Pressure-dependent changes in haematocrit and plasma volume during anaesthesia, a randomised clinical trial. *Acta Anaesthesiol Scand*. 2016;60(5):560-8.

21. Urzua J, Troncoso S, Bugedo G, Canessa R, Muñoz H, Lema G, et al. Renal function and cardiopulmonary bypass: effect of perfusion pressure. *J Cardiothorac Vasc Anesth*. 1992;6(3):299-303.

22. Bagheri K, Motamedi O, Aghadavoudi O, Akbari MJJoIMS. The Effects of Mean Arterial Pressure during Cardiopulmonary Bypass on Clinical and Paraclinical Parameters during and after Coronary Artery Bypass Graft Surgery. 2012;29(169).

23. Charlson ME, Peterson JC, Krieger KH, Hartman GS, Hollenberg JP, Briggs WM, et al. Improvement of outcomes after coronary artery bypass II: a randomized trial comparing intraoperative high versus customized mean arterial pressure. 2007;22(6):465-72.

24. Suk P, Leverve X, Hruda J, Sramek VJS. EARLY RESUSCITATION OF SEPTIC SHOCK TO DIFFERENT LEVEL OF ARTERIAL PRESSURE. 2006;26(4):38.

25. Maiwall R, editor High Versus Low Target Mean Arterial Pressure in Septic Shock in Critically Ill Cirrhotics. AASLD The Liver Meeting; 2021 10:00 - 11:30 AM EST, Monday, November 15th, 2021; Parallel 25: Portal Hypertension Clinical.

26. Grand J, Meyer AS, Kjaergaard J, Wiberg S, Thomsen JH, Frydland M, et al. A randomised double-blind pilot trial comparing a mean arterial pressure target of 65 mm Hg versus 72 mm Hg after out-of-hospital cardiac arrest. *Eur Heart J Acute Cardiovasc Care*. 2020;9(4_suppl):S100-s9.

27. Lamontagne F, Richards-Belle A, Thomas K, Harrison DA, Sadique MZ, Grieve RD, et al. Effect of Reduced Exposure to Vasopressors on 90-Day Mortality in Older Critically Ill Patients With Vasodilatory Hypotension: A Randomized Clinical Trial. *Jama*. 2020;323(10):938-49.

28. Futier E, Lefrant JY, Guinot PG, Godet T, Lorne E, Cuvillon P, et al. Effect of Individualized vs Standard Blood Pressure Management Strategies on Postoperative Organ Dysfunction Among High-Risk Patients Undergoing Major Surgery: A Randomized Clinical Trial. *Jama*. 2017;318(14):1346-57.

29. Hu AM, Qiu Y, Zhang P, Zhao R, Li ST, Zhang YX, et al. Higher versus lower mean arterial pressure target management in older patients having non-cardiothoracic surgery: A prospective randomized controlled trial. *J Clin Anesth*. 2021;69:110150.

30. Wanner PM, Wulff DU, Djurdjevic M, Korte W, Schnider TW, Filipovic M. Targeting Higher Intraoperative Blood Pressures Does Not Reduce Adverse Cardiovascular Events Following Noncardiac Surgery. *J Am Coll Cardiol*. 2021;78(18):1753-64.

31. Wu X, Jiang Z, Ying J, Han Y, Chen Z. Optimal blood pressure decreases acute kidney injury after gastrointestinal surgery in elderly hypertensive patients: A randomized study: Optimal blood pressure reduces acute kidney injury. *J Clin Anesth*. 2017;43:77-83.

32. Azau A, Markowicz P, Corbeau JJ, Cottineau C, Moreau X, Baufreton C, et al. Increasing mean arterial pressure during cardiac surgery does not reduce the rate of postoperative acute kidney injury. *Perfusion*. 2014;29(6):496-504.

33. Kandler K, Nilsson JC, Oturai P, Jensen ME, Møller CH, Clemmesen JO, et al. Higher arterial pressure during cardiopulmonary bypass may not reduce the risk of acute kidney injury. *J Cardiothorac Surg*. 2019;14(1):107.

34. Siepe M, Pfeiffer T, Gieringer A, Zemann S, Benk C, Schlensak C, et al. Increased systemic perfusion pressure during cardiopulmonary bypass is associated with less early postoperative cognitive dysfunction and delirium. *Eur J Cardiothorac Surg*. 2011;40(1):200-7.

35. Vedel AG, Holmgaard F, Rasmussen LS, Langkilde A, Paulson OB, Lange T, et al. High-Target Versus Low-Target Blood Pressure Management During Cardiopulmonary Bypass to Prevent Cerebral Injury in Cardiac Surgery Patients: A Randomized Controlled Trial. *Circulation*. 2018;137(17):1770-80.
